# Prediction of enteric methane production, yield, and intensity in dairy cattle using an intercontinental database

**DOI:** 10.1111/gcb.14094

**Published:** 2018-03-08

**Authors:** Mutian Niu, Ermias Kebreab, Alexander N. Hristov, Joonpyo Oh, Claudia Arndt, André Bannink, Ali R. Bayat, André F. Brito, Tommy Boland, David Casper, Les A. Crompton, Jan Dijkstra, Maguy A. Eugène, Phil C. Garnsworthy, Md Najmul Haque, Anne L. F. Hellwing, Pekka Huhtanen, Michael Kreuzer, Bjoern Kuhla, Peter Lund, Jørgen Madsen, Cécile Martin, Shelby C. McClelland, Mark McGee, Peter J. Moate, Stefan Muetzel, Camila Muñoz, Padraig O'Kiely, Nico Peiren, Christopher K. Reynolds, Angela Schwarm, Kevin J. Shingfield, Tonje M. Storlien, Martin R. Weisbjerg, David R. Yáñez‐Ruiz, Zhongtang Yu

**Affiliations:** ^1^ Department of Animal Science University of California Davis CA USA; ^2^ Department of Animal Science The Pennsylvania State University University Park PA USA; ^3^ Environmental Defense Fund San Francisco CA USA; ^4^ Wageningen Livestock Research Wageningen University & Research Wageningen The Netherlands; ^5^ Milk Production Solutions, Green Technology Natural Resources Institute Finland (Luke) Jokioinen Finland; ^6^ Department of Agriculture, Nutrition and Food Systems University of New Hampshire Durham NH USA; ^7^ School of Agriculture and Food Science University College Dublin Belfield, Dublin 4 Ireland; ^8^ Furst McNess Company Freeport IL USA; ^9^ School of Agriculture, Policy and Development University of Reading Reading UK; ^10^ Animal Nutrition Group Wageningen University & Research Wageningen The Netherlands; ^11^ UMR Herbivores, INRA, VetAgro Sup, Université Clermont Auvergne Saint‐Genès‐Champanelle France; ^12^ School of Biosciences University of Nottingham Loughborough UK; ^13^ Department of Large Animal Sciences University of Copenhagen Copenhagen Denmark; ^14^ Department of Animal Science Aarhus University Tjele Denmark; ^15^ Department of Agricultural Science for Northern Sweden Swedish University of Agricultural Sciences Umeå Sweden; ^16^ ETH Zurich Institute of Agricultural Sciences Zurich Switzerland; ^17^ Institute of Nutritional Physiology Leibniz Institute for Farm Animal Biology Dummerstorf Mecklenburg‐Vorpommern Germany; ^18^ Department of Soil and Crop Sciences Colorado State University Fort Collins CO USA; ^19^ Teagasc, Agriculture and Food Development Authority Carlow Ireland; ^20^ Agriculture Research Division Department of Economic Development, Jobs, Transport and Resources Melbourne Vic. Australia; ^21^ Ag Research Palmerston North New Zealand; ^22^ Instituto de Investigaciones Agropecuarias, INIA Remehue Osorno Chile; ^23^ Animal Sciences Department Flanders Research Institute for Agriculture Fisheries and Food Melle Belgium; ^24^ Institute of Biological, Environmental and Rural Sciences Aberystwyth University Aberystwyth UK; ^25^ Department of Animal and Aquacultural Sciences Norwegian University of Life Sciences Ås Norway; ^26^ Estación Experimental del Zaidin (CSIC) Granada Spain; ^27^ Department of Animal Sciences The Ohio State University Columbus OH USA

**Keywords:** dairy cows, dry matter intake, enteric methane emissions, methane intensity, methane yield, prediction models

## Abstract

Enteric methane (CH_4_) production from cattle contributes to global greenhouse gas emissions. Measurement of enteric CH_4_ is complex, expensive, and impractical at large scales; therefore, models are commonly used to predict CH_4_ production. However, building robust prediction models requires extensive data from animals under different management systems worldwide. The objectives of this study were to (1) collate a global database of enteric CH_4_ production from individual lactating dairy cattle; (2) determine the availability of key variables for predicting enteric CH_4_ production (g/day per cow), yield [g/kg dry matter intake (DMI)], and intensity (g/kg energy corrected milk) and their respective relationships; (3) develop intercontinental and regional models and cross‐validate their performance; and (4) assess the trade‐off between availability of on‐farm inputs and CH_4_ prediction accuracy. The intercontinental database covered Europe (EU), the United States (US), and Australia (AU). A sequential approach was taken by incrementally adding key variables to develop models with increasing complexity. Methane emissions were predicted by fitting linear mixed models. Within model categories, an intercontinental model with the most available independent variables performed best with root mean square prediction error (RMSPE) as a percentage of mean observed value of 16.6%, 14.7%, and 19.8% for intercontinental, EU, and United States regions, respectively. Less complex models requiring only DMI had predictive ability comparable to complex models. Enteric CH_4_ production, yield, and intensity prediction models developed on an intercontinental basis had similar performance across regions, however, intercepts and slopes were different with implications for prediction. Revised CH_4_ emission conversion factors for specific regions are required to improve CH_4_ production estimates in national inventories. In conclusion, information on DMI is required for good prediction, and other factors such as dietary neutral detergent fiber (NDF) concentration, improve the prediction. For enteric CH_4_ yield and intensity prediction, information on milk yield and composition is required for better estimation.

## 1 INTRODUCTION

Emissions of greenhouse gases (GHG) have a considerable impact on climate change, which is an ongoing threat for global food security. Global food demand in the next 30 years is projected to increase by over 60% compared to 2006, with more than 321 million people worldwide at risk of hunger without implementation of climate change mitigation policies (FAO, 2016). The GHG emissions from livestock are estimated to be 7.1 Gt carbon dioxide (CO_2_) equivalents per year accounting for 14.5% of global anthropogenic GHG emissions (Gerber et al., 2013). Methane (CH_4_) is emitted from livestock mainly through enteric fermentation and manure decomposition. Enteric CH_4_ is a natural by‐product of microbial fermentation of nutrients in the digestive tract of animals. Globally, most attention has been directed to enteric CH_4_ emissions from ruminants, particularly cattle because these farm species have been shown to be the major contributors of total GHG emissions from the livestock sector (Gerber et al., 2013; Tubiello et al., 2013).

Attempts to reduce the carbon footprint of animal agriculture systems, primarily on‐farm GHG emissions, will ideally involve implementation of mitigation strategies without compromising animal productivity or social acceptability, and without endangering animal health or welfare. To reduce the impact on the environment, the amount of CH_4_ produced within a production system needs to be quantified accurately so that emission can be mitigated through management. Direct measurement of enteric CH_4_ production from cattle can be conducted using various techniques including several bottom–up and some top–down approaches, i.e., based on national or regional activity data and emission factors for different CH_4_ sources, or on atmospheric measurements, respectively. However, measurements of CH_4_ production from individual animals, groups of animals, or at a regional level is expensive and requires specialized equipment (Hammond et al., 2016; Kebreab, Clark, Wagner‐Riddle, & France, 2006). Proxies (i.e., indicators or indirect traits) for CH_4_ emissions have also been qualitatively explored, but no single proxy was found to accurately predict CH_4_ and combinations of proxies to date are not sufficiently robust for general applicability (Negussie et al., 2017). Therefore, quantitative approaches such as mathematical modeling have been used to estimate CH_4_ production in cattle (Kebreab, Johnson, Archibeque, Pape, & Wirth, 2008). Both mechanistic and empirical approaches have been used to predict enteric CH_4_ emissions. However, mechanistic models are usually more detailed and require numerous inputs that may not be readily available; therefore, their utility in practice is reduced. An empirical prediction approach requires fewer inputs and can generally be implemented by a much wider audience including scientists and policy makers. There are over 40 empirical prediction equations for enteric CH_4_ production of lactating dairy cows in the literature (Appuhamy, France, & Kebreab, 2016). The majority of these models were based on measurements from relatively small numbers of animals in the same geographic region, which may limit their application in other regions. Therefore, a more comprehensive database needs to be collated to develop enteric CH_4_ production prediction models at both global and regional scales. Furthermore, the performance of global models in each geographic region should be evaluated and compared with regional‐specific prediction models.

The CH_4_ conversion factor (*Y*_m_) was introduced by the Intergovernmental Panel on Climate Change (IPCC) to indicate the proportion of the animal's gross energy intake (GEI) converted to enteric CH_4_ energy, and it is widely used for national GHG emission inventories and global research on mitigation strategies. However, it has been consistently shown that CH_4_ emissions are related not only to feed intake but also to feed nutrient compositions, which *Y*_m_‐based models cannot adequately represent (Ellis, Bannink, France, Kebreab, & Dijkstra, 2010). Therefore, identifying relationships between dietary variables and CH_4_ production and their impacts on prediction and model performance are critical. Several extant prediction models require inputs that may not be commonly available in a commercial dairy production system. Although predictive ability is likely to be enhanced with model complexity (Moraes, Strathe, Fadel, Casper, & Kebreab, 2014; Santiago‐Juarez et al., 2016), the trade‐off between availability of variable inputs on farm and prediction accuracy of enteric CH_4_ production of dairy cows must be carefully considered. This is because more complex models may contain predictor variables that are expensive and not easy to obtain and thereby not applicable, especially in developing countries. Therefore, a categorization of model types which reflect different types and levels of data availability (e.g., diet composition, milk production and composition, and animal characteristics) needs to be conducted. Evaluation of model performance across various categories can be useful for different groups (e.g., researchers, regulators etc.).

The objectives of this study were to: (1) collate a global database of enteric CH_4_ production in individual lactating dairy cows; (2) determine the availability of key variables for predicting enteric CH_4_ production (g/day per cow), yield [g/kg dry matter intake (DMI)], and intensity [g/kg energy corrected milk (ECM)] and their respective relationships; (3) develop intercontinental and regional‐specific prediction equations for CH_4_ production, yield, and intensity using a large individual cow database and cross‐validate their performance; and (4) assess the trade‐off between the availability of on‐farm variable inputs and prediction accuracy of enteric CH_4_ production, yield, and intensity in lactating dairy cows.

## 2 MATERIALS AND METHODS

### 2.1 Database

The “GLOBAL NETWORK” project (Global Network for the Development and Maintenance of Nutrition‐Related Strategies for Mitigation of Methane and Nitrous Oxide Emissions from Ruminant Livestock; 2014–2018) is an international collaborative initiative of animal scientists from all continents, except Africa (http://animalscience.psu.edu/fnn; accessed May 16, 2017). The dairy CH_4_ database, developed in the frame of the “GLOBAL NETWORK” project, contains 5,233 individual dairy cow records from 154 published and unpublished studies conducted from 1962 to 2016 by researchers and research institutes from 15 countries in Europe (EU; *n* = 3,015 from 82 studies), the United States of America (US; *n* = 1,916 from 64 studies), Chile (CL; *n* = 108 from 3 studies), Australia (AU; *n* = 64 from 1 study), and New Zealand (NZ; *n* = 130 from 4 studies). The database includes records of enteric CH_4_ production along with corresponding DMI, dietary concentration of gross energy (GE), crude protein (CP), ether extract (EE), neutral detergent fiber (NDF), and ash. It also includes milk yield (MY), concentrations of milk fat (MF) and crude protein (MP), and body weight (BW) records. The EU studies were conducted in the United Kingdom (*n* = 930 from 38 studies), Denmark (*n* = 512 from 12 studies), Switzerland (*n* = 483 from 9 studies), Sweden (*n* = 357 from 5 studies), the Netherlands (*n* = 188 from 5 studies), Finland (*n* = 170 from 2 studies), Belgium (*n* = 104 from 4 studies), Ireland (*n* = 90 from 1 study), Norway (*n* = 88 from 4 studies), Germany (*n* = 61 from 1 study), and France (*n* = 32 from 1 study).

Energy corrected milk (3.5% fat) was calculated based on an equation derived from Tyrrell and Reid (1965): ECM (kg/day) = 12.95 × fat yield (kg/day) + 7.65 × true protein yield (kg/day; i.e., crude protein × 0.93) + 0.327 × milk yield (kg/day). The majority of studies had measured GE. If the feed ingredients and proportions in the diets were known, the GE was calculated from book values (about 6%). Methane yield (CH_4_ production divide by DMI) and intensity (CH_4_ production divide by ECM) were calculated for all records.

The majority of the studies in the database had investigated the impact of diet composition on enteric CH_4_ production. However, about 20% of the studies tested the effect of feed additives or pure nutrient supplementation, so data from these studies were either completely excluded or only the control treatments were retained. These feed additives included nitrate (Olijhoek et al., 2016), 3‐nitrooxypropanol (Hristov et al., 2015), and intragastric infusion of acetate, propionate, glucose, and *cis*‐ or *trans‐*fatty acids. Measurements of enteric CH_4_ production were conducted using various approaches although the observations from a given research group were usually measured using the same approach. To ensure data quality, only enteric CH_4_ measurements from respiration chambers, the GreenFeed system (C‐Lock Inc., Rapid City, SD), and sulfur hexafluoride (SF_6_) tracer technique were retained for the analysis.

The variable selection and model evaluation approaches required complete data for all predictor and response variables. Therefore, records missing any predictor or response variable information were removed before being screened for outliers. Outliers in the database were screened using the interquartile range (IQR) method (Zwillinger & Kokoska, 2000) based on CH_4_ yield and intensity records for each region. In this study, a factor of 1.5 for extremes was used in constructing markers to identify outliers, as shown in the Equations (i), (ii), (iii):

(i) IQR=Third Quartile (Q3)−First Quartile (Q1);

(ii) Lower Fence=Q1−(IQR×1.5);

(iii) Upper Fence=Q3+(IQR×1.5).

As a result, a refined complete data set (*n* = 2,566), containing complete information on CH_4_ production, DMI, GEI, dietary concentrations of GE, CP, EE, NDF, and ash, MY, MF, MP, ECM, and BW were used for variable selection and comparison of prediction model performance for lactating dairy cows as described below. Summary statistics for EU, the United States, and intercontinental records (combination of EU, US, and AU data) are shown in Table 1. Overall, the data set comprised individual observations from Holstein (68%; *n* = 1,732), Ayrshire (19%; *n* = 497), Jersey (3%; *n* = 88), as well as Brown Swiss, Simmental, and crossbred dairy cattle (a total of 10%; *n* = 249). The breakdown of observations in the complete intercontinental data set was 1,423 from EU (42 studies), 1,084 from the United States (45 studies), and 59 from AU (1 study). Ninety‐one percent of the US observations were from Holstein cows, while the remaining were Jersey. Holstein, Ayrshire, Jersey, and other breeds provided 44, 35, 3, and 18% of EU data, respectively, and AU data were from Holstein cows.

**Table 1:** Summary statistics of the refined complete data set in different regions

Item^a^	Intercontinental^b^ (*n* = 2,566)				EU (*n* = 1,423)				US (*n* = 1,084)			
	Mean	Min^c^	Max	*SD*	Mean	Min	Max	*SD*	Mean	Min	Max	*SD*
DMI (kg/day)	18.5	3.9	35.4	4.60	18.5	8.0	33.5	3.84	18.8	3.9	35.4	5.48
GEI (MJ/day)	347	75	644	89.3	345	137	606	73.5	354	75	644	103.1
Diet composition (% of DM)												
CP	16.5	8.1	25.3	2.43	16.5	8.1	25.3	2.58	16.5	9.8	23.5	2.18
EE	3.5	0.7	7.7	1.14	3.6	1.5	7.7	1.06	3.3	0.7	7.0	1.23
ash	7.3	3.4	19.5	1.76	7.9	3.7	19.5	1.89	6.4	3.4	12.1	1.07
NDF	35.4	13.4	70.0	7.66	36.6	13.4	57.0	7.83	33.3	14.9	70.0	6.77
GE (MJ/kg DM)	18.7	16.1	22.8	0.69	18.6	16.1	22.8	0.75	18.8	17.3	20.7	0.56
Yield												
MY, kg/day	27.0	4.3	62.7	9.76	26.4	7.6	51.4	7.92	28.4	4.3	62.7	11.50
ECM, kg	29.2	5.5	64.6	9.78	29.8	11.4	56.3	8.05	29.0	5.5	64.6	11.55
Milk composition (%)												
MF	4.1	1.4	9.0	0.85	4.4	1.8	9.0	0.80	3.6	1.4	7.6	0.68
MP	3.4	2.3	5.3	0.38	3.4	2.3	4.9	0.37	3.2	2.3	5.3	0.35
BW (kg)	611	283	939	88.1	614	283	939	89.3	611	302	854	86.4
Methane emissions												
CH_4_ (g/day per cow)	369	79	729	100.7	392	169	701	88.8	340	79	729	109.3
CH_4_/DMI (g/kg)	20.1	9.0	30.4	3.87	21.4	12.3	30.4	3.39	18.2	9.0	28.0	3.71
CH_4_/ECM (g/kg)	13.5	3.0	36.0	3.92	13.6	5.1	22.3	3.07	12.8	3.0	24.8	4.25
*Y*_m_ d (% of GEI)	6.0	2.7	9.8	1.18	6.4	3.6	9.8	1.04	5.4	2.7	8.4	1.09

a DM, dry matter; DMI, dry matter intake; GEI, gross energy intake; CP, dietary crude protein concentration; EE, dietary ether extract concentration; ash, dietary ash concentration; NDF, dietary neutral detergent fiber concentration; MY, milk yield; ECM, energy corrected milk; MF, milk fat concentration; MP, milk crude protein concentration; BW, body weight.

b EU, Europe; US, the United States of America; AU, Australia; Intercontinental = (EU + US + AU).

c Min, minimum; Max, maximum; *SD*, standard deviation.

d Methane conversion factor (%) = energy of CH_4_ as a percentage of GEI.

Unlike variable selection and model evaluation approaches, the construction of equations does not require data that contain a complete set of all predictor variables. Therefore, to be able to maximize the amount of data useful to construct prediction equations, subsets of data that contain complete information of CH_4_ production and of the selected corresponding predictor variables were screened for outliers using the IQR method stated above and then used for the construction of equations. The same approaches were done for CH_4_ yield and intensity models.

### 2.2 Model development

Model development was conducted using a sequential approach, by incrementally adding different levels (e.g., dietary composition, milk production and composition, and animal traits) of variables to develop models with increasing complexity. In total, 11 CH_4_ production prediction models with different complexity categories were developed (Table 2) using the following information: GEI only (GEI_C), DMI only (DMI_C), DMI and dietary NDF concentration (DMI + NDF_C), DMI and dietary EE concentration (DMI + EE_C), DMI and all dietary composition (DMI + Com_C), all available dietary composition only (Diet_Com_C), MY only (MY_C), ECM only (ECM_C), ECM and milk composition (ECM + Com_C), all the available variables (Animal_C), and all available variables except DMI (Animal_no_DMI_C). Within each category, both intercontinental and regional models were developed; however, regional models were for EU and United States only due to the limited number of observations from other regions (Tables 3 and 4). Seven CH_4_ yield models with different complexity levels were developed (Table 5) without predictors associated with DMI (Table 5). The categories were: dietary NDF concentration (NDF_C), dietary EE concentration (EE_C), Diet_Com_C, MY_C, ECM_C, ECM + Com_C, and Animal_no_DMI_C. Similarly, 9 CH_4_ intensity models with different complexity levels were developed (Table 6) without using either MY or ECM: GEI_C, DMI_C, DMI + NDF_C, DMI + EE_C, DMI + Com_C, Diet_Com_C, milk composition only (Milk_Com_C), all the available variables except MY and ECM (Animal_C), and all available variables except DMI, MY, and ECM (Animal_no_DMI_C) (Table 6). As described above, the refined complete data set (*n* = 2,566) that contains all predictor variables was used for model selection and evaluation, and the final equation was constructed based on a complete data set that only contained the selected predictor variables of the corresponding model for each category. Therefore, the amount of data was maximized for the development of equations at different complexity levels.

**Table 2:** Intercontinental CH_4_ production (g/day per cow) prediction equations for various complexity levels and model evaluations across regions

Model development				Model performance^d^					
Equation	Category	Prediction equation^a^	*n* b	Region^c^	RMSPE, %	RSR	MB, %	SB, %	CCC
(1)	GEI_C	[7.13 (0.581) + 0.0391 (0.00095) × GEI]/0.05565	3,352	Intercontinental	17.8	0.65	0.99	0.11	0.72
				EU	15.9	0.70	5.02	1.44	0.66
				US	20.8	0.65	0.49	0.65	0.75
(2)	DMI_C	124 (10.44) + 13.3 (0.32) × DMI	3,384	Intercontinental	17.5	0.64	1.09	0.27	0.73
				EU	15.2	0.67	5.42	2.98	0.69
				US	21.0	0.65	0.36	0.63	0.74
(3)	DMI + NDF_C	33.2 (13.54) + 13.6 (0.33) × DMI + 2.43 (0.245) × NDF	3,116	Intercontinental	17.1	0.63	0.75	0.44	0.75
				EU	14.8	0.65	3.70	4.11	0.70
				US	20.5	0.64	0.11	0.50	0.76
(4)	DMI + EE_C	163 (12.9) + 13.3 (0.35) × DMI − 11.0 (1.39) × EE	2,716	Intercontinental	17.7	0.65	1.15	0.52	0.72
				EU	15.2	0.67	4.96	3.33	0.69
				US	21.5	0.67	0.14	0.25	0.72
(5)	DMI + Com_C	76.0 (16.14) + 13.5 (0.35) × DMI − 9.55 (1.390) × EE + 2.24 (0.268) × NDF	2,667	Intercontinental	17.3	0.63	0.79	0.71	0.74
				EU	14.9	0.66	3.29	3.69	0.70
				US	20.8	0.65	0.02	0.10	0.74
(6)	Diet_Com_C	369 (21.9) − 14.7 (1.73) × EE + 1.67 (0.339) × NDF	2,667	Intercontinental	25.2	0.92	0.56	1.95	0.34
				EU	22.0	0.97	0.44	1.13	0.18
				US	30.0	0.93	1.13	2.54	0.34
(7)	MY_C	299 (12.1) + 2.73 (0.171) × MY	3,384	Intercontinental	21.7	0.80	0.58	0.69	0.51
				EU	19.0	0.84	1.60	7.62	0.39
				US	25.9	0.80	0	0.11	0.53
(8)	ECM_C	259 (11.1) + 3.86 (0.167) × ECM	3,384	Intercontinental	20.3	0.74	0.49	0.96	0.59
				EU	17.5	0.77	1.38	8.93	0.51
				US	24.4	0.76	0	0.06	0.60
(9)	ECM + Com_C	150 (16.1) + 4.31 (0.172) × ECM + 28.3 (3.20) × MP	3,384	Intercontinental	19.8	0.72	0.55	1.16	0.62
				EU	16.9	0.75	1.32	9.28	0.55
				US	23.8	0.74	0	0.01	0.62
(10)	Animal_C	−60.5 (17.56) + 12.4 (0.37) × DMI − 8.78 (1.342) × EE + 2.10 (0.256) × NDF + 16.1 (1.39) × MF + 0.148 (0.0143) × BW	2,566	Intercontinental	16.6	0.61	0.91	1.51	0.76
				EU	14.7	0.64	2.83	4.48	0.72
				US	19.8	0.62	0.02	0.08	0.76
(11)	Animal_no_DMI_C	−37.0 (22.94) − 12.3 (1.49) × EE + 2.24 (0.289) × NDF + 3.68 (0.191) × ECM + 7.81 (1.762) × MF + 17.7 (3.78) × MP + 0.284 (0.0148) × BW	2,566	Intercontinental	18.9	0.69	0.62	1.04	0.66
				EU	15.9	0.70	0.84	5.57	0.63
				US	23.2	0.72	0.35	0.08	0.64
(12)	IPCC, 2006, e	(0.065 × GEI)/0.05565	‐	Intercontinental	22.8	0.84	18.8	12.3	0.68
				EU	16.2	0.71	2.87	9.63	0.74
				US	31.5	0.98	48.1	10.9	0.64
(13)	IPCC, 1997	(0.060 × GEI)/0.05565	‐	Intercontinental	19.9	0.73	0.53	8.70	0.72
				EU	16.3	0.72	10.0	4.17	0.72
				US	24.9	0.77	25.1	9.44	0.73

a GEI, gross energy intake (MJ/day); DMI, dry matter intake (kg/day); NDF, dietary neutral detergent fiber concentration (% of DM); EE, dietary ether extract concentration (% of DM); MY, milk yield (kg/day); ECM, energy corrected milk (kg/day); MF, milk fat concentration (%); MP, milk crude protein concentration (%); BW, body weight (kg).

b *n*, number of observations used to construct equations.

c EU, Europe; US, the United State of America; AU, Australia. Number of observations used for model performance cross‐validation: Intercontinental (EU + US + AU; *n* = 2,566); EU (*n* = 1,423); US (*n* = 1,084).

d RMSPE, Root mean square prediction error, expressed as a percentage of observed CH_4_ production means; RSR, RMSPE‐observations standard deviation ratio; MB, mean bias as a percentage of MSPE, SB, slope bias as a percentage of MSPE; CCC, Concordance Correlation Coefficient.

e IPCC, Intergovernmental Panel on Climate Change. Mean CH_4_ production prediction of IPCC, 2006 model is 406, 402, and 414 g/day per cow for Intercontinental, EU, and US cows, respectively; mean CH_4_ production prediction of IPCC, 1997 model is 374, 371, and 382 g/day per cow for Intercontinental, EU, and US cows, respectively. The observed mean CH_4_ production is 369, 392, and 340 g/day per cow for Intercontinental, EU, and US cows, respectively (Table 1).

**Table 3:** Europe (EU) CH_4_ production (g/day per cow) prediction equations for various complexity levels and model evaluations across regions

Model development				Model performance^d^					
Equation	Category	Prediction equation^a^	*n* b	Region^c^	RMSPE, %	RSR	MB, %	SB, %	CCC
(14)	GEI_C	[6.20 (0.688) + 0.0425 (0.00118) × GEI]/0.05565	1,990	Intercontinental	19.1	0.70	2.23	0.28	0.67
				EU	15.9	0.70	3.79	0.54	0.67
				US	23.8	0.74	27.9	0.45	0.68
(15)	DMI_C	107 (12.6) + 14.5 (0.39) × DMI	2,022	Intercontinental	18.4	0.67	1.86	0.52	0.70
				EU	15.0	0.66	3.72	1.27	0.71
				US	23.3	0.72	24.1	0.26	0.69
(16)	DMI + NDF_C	−26.0 (16.67) + 15.3 (0.41) × DMI + 3.42 (0.309) × NDF	1,779	Intercontinental	17.9	0.66	2.12	0.42	0.72
				EU	14.7	0.65	1.63	1.05	0.72
				US	22.4	0.70	18.6	0	0.71
(17)	DMI + EE_C	160 (14.7) + 14.2 (0.44) × DMI − 13.5 (1.46) × EE	1,516	Intercontinental	19.1	0.70	2.62	1.70	0.65
				EU	15.1	0.67	3.32	1.29	0.70
				US	24.7	0.77	25.9	2.94	0.62
(18)	DMI + Com_C	11.3 (22.62) + 14.7 (0.44) × DMI + 2.50 (0.670) × CP − 10.8 (1.49) × EE + 3.20 (0.361) × NDF − 2.87 (1.134) × ash	1,467	Intercontinental	18.8	0.69	4.50	1.47	0.68
				EU	14.7	0.65	1.40	1.58	0.71
				US	24.4	0.76	28.2	1.36	0.65
(19)	Diet_Com_C	435 (17.4) − 18.7 (1.92) × EE	1,467	Intercontinental	28.4	1.04	0.64	7.43	0.01
				EU	22.0	0.97	1.41	1.31	0.20
				US	37.5	1.16	6.72	40.0	‐0.20
(20)	MY_C	287 (14.1) + 3.16 (0.224) × MY	2,022	Intercontinental	22.8	0.83	1.70	4.69	0.41
				EU	18.4	0.81	1.15	3.67	0.45
				US	28.8	0.90	15.2	6.49	0.37
(21)	ECM_C	247 (13.1) + 4.30 (0.215) × ECM	2,022	Intercontinental	21.0	0.77	1.12	5.04	0.53
				EU	17.2	0.76	1.04	4.48	0.55
				US	26.2	0.81	12.1	5.18	0.50
(22)	ECM + Com_C	141 (18.9) + 4.75 (0.220) × ECM + 27.4 (3.70) × MP	2,022	Intercontinental	20.1	0.74	0.74	5.00	0.58
				EU	16.6	0.73	0.99	4.64	0.58
				US	25.0	0.78	9.56	4.45	0.55
(23)	Animal_C	−52.2 (21.73) + 13.0 (0.49) × DMI − 10.9 (1.50) × EE + 2.80 (0.349) × NDF + 7.26 (1.590) × MF + 0.154 (0.0167) × BW	1,423	Intercontinental	17.7	0.65	1.42	4.57	0.70
				EU	14.6	0.64	2.58	2.60	0.72
				US	22.3	0.69	16.8	4.58	0.68
(24)	Animal_no_DMI_C	−44.7 (27.14) − 15.3 (1.63) × EE + 2.62 (0.391) × NDF + 4.34 (0.242) × ECM + 21.5 (3.83) × MP + 0.289 (0.0168) × BW	1,423	Intercontinental	20.0	0.73	0.54	4.35	0.59
				EU	15.8	0.70	1.43	2.11	0.65
				US	25.7	0.80	7.70	5.81	0.50

a GEI, gross energy intake (MJ/day); DMI, dry matter intake (kg/day); NDF, dietary neutral detergent fiber concentration (% of DM); EE, dietary ether extract concentration (% of DM); ash, dietary ash concentration (% of DM); MY, milk yield (kg/day); ECM, energy corrected milk (kg/day); MF, milk fat concentration (%); MP, milk crude protein concentration (%); BW, body weight (kg).

b *n*, number of observations used to construct equations

c EU, Europe; US, the United States of America; AU, Australia. Number of observations used for model performance cross‐validation: Intercontinental (EU + US + AU; *n* = 2,566); EU (*n* = 1,423); US (*n* = 1,084).

d RMSPE, Root mean square prediction error, expressed as a percentage of observed CH_4_ production means; RSR, RMSPE‐observations standard deviation ratio; MB, mean bias as a percentage of MSPE, SB, slope bias as a percentage of MSPE; CCC, Concordance Correlation Coefficient.

**Table 4:** The US CH_4_ production (g/day per cow) prediction equations for various complexity levels and model evaluations across regions

Model development				Model performance^d^					
Equation	Model	Prediction equation^a^	*n* b	Region^c^	RMSPE, %	RSR	MB, %	SB, %	CCC
(25)	GEI_C	[7.30 (1.217) + 0.0358 (0.00163) × GEI]/0.05565	1,212	Intercontinental	19.5	0.71	8.80	1.07	0.66
				EU	18.5	0.81	27.4	5.60	0.55
				US	21.0	0.65	0.09	0.09	0.73
(26)	DMI_C	125 (20.5) + 12.2 (0.55) × DMI	1,212	Intercontinental	19.8	0.73	10.4	1.21	0.64
				EU	18.8	0.83	30.9	5.93	0.54
				US	21.3	0.66	0.02	0.03	0.72
(27)	DMI + NDF_C	49.5 (27.78) + 12.1 (0.56) × DMI + 2.57 (0.450) × NDF	1,187	Intercontinental	18.4	0.67	5.49	2.11	0.69
				EU	16.6	0.73	18.1	10.3	0.62
				US	21.1	0.65	0	0.07	0.73
(28)	DMI + EE_C	136 (27.1) + 12.3 (0.57) × DMI − 2.96 (2.876) × EE	1,141	Intercontinental	19.8	0.72	9.85	1.37	0.64
				EU	18.7	0.82	29.8	6.57	0.55
				US	21.4	0.67	0.02	0.03	0.72
(29)	DMI + Com_C	49.5 (27.78) + 12.1 (0.56) × DMI + 2.57 (0.450) × NDF	1,187	Intercontinental	18.4	0.67	5.49	2.11	0.69
				EU	16.6	0.73	18.1	10.3	0.62
				US	21.1	0.65	0	0.07	0.73
(30)	Diet_Com_C	279 (51.1) + 3.53 (0.531) × NDF	1,141	Intercontinental	25.6	0.94	0.93	5.07	0.38
				EU	23.3	1.03	3.74	3.27	0.08
				US	28.8	0.89	0.29	3.62	0.44
(31)	MY_C	314 (33.4) + 2.27 (0.278) × MY	1,212	Intercontinental	23.0	0.84	1.36	0.33	0.43
				EU	20.9	0.92	4.11	14.7	0.21
				US	26.5	0.82	0.01	0.59	0.51
(32)	ECM_C	270 (28.9) + 3.44 (0.278) × ECM	1,212	Intercontinental	21.5	0.79	1.71	1.05	0.53
				EU	19.3	0.85	5.27	13.8	0.37
				US	24.8	0.77	0	0.23	0.59
(33)	ECM + Com_C	157 (37.1) + 3.53 (0.295) × ECM + 16.1 (3.22) × MF + 15.3 (6.83) × MP	1,212	Intercontinental	20.8	0.76	0.04	0.55	0.57
				EU	18.6	0.82	0.05	11.2	0.40
				US	24.3	0.75	0.01	0.14	0.61
(34)	Animal_C	−126 (32.7) + 11.3 (0.59) × DMI + 2.30 (0.414) × NDF + 28.8 (2.53) × MF + 0.148 (0.0250) × BW	1,084	Intercontinental	16.8	0.62	0.47	1.36	0.75
				EU	14.9	0.66	1.57	8.21	0.68
				US	19.8	0.62	0.01	0.01	0.77
(35)	Animal_no_DMI_C	−72.4 (42.41) + 3.15 (0.461) × NDF + 2.65 (0.270) × ECM + 23.9 (2.79) × MF + 0.290 (0.0257) × BW	1,084	Intercontinental	19.9	0.73	0.80	0.01	0.64
				EU	17.7	0.78	3.33	4.18	0.52
				US	21.1	0.72	0.08	0.23	0.66

a GEI, gross energy intake (MJ/day); DMI, dry matter intake (kg/day); NDF, dietary neutral detergent fiber concentration (% of DM); EE, dietary ether extract concentration (% of DM); MY, milk yield (kg/day); ECM, energy corrected milk (kg/day); MF, milk fat concentration (%); MP, milk crude protein concentration (%); BW, body weight (kg).

b *n*, number of observations used to construct equations.

c EU, Europe; US, the United States of America; AU, Australia. Number of observations used for model performance cross‐validation: Intercontinental (EU + US + AU; *n* = 2,566); EU (*n* = 1,423); US (*n* = 1,084).

d RMSPE, Root mean square prediction error, expressed as a percentage of observed CH_4_ production means; RSR, RMSPE‐observations standard deviation ratio; MB, mean bias as a percentage of MSPE, SB, slope bias as a percentage of MSPE; CCC, Concordance Correlation Coefficient.

**Table 5:** Intercontinental CH_4_ yield prediction (g/kg DMI) prediction equations and model evaluations across regions

Model development				Model performance^d^					
Equation	Category	Prediction equation^a^	*n* b	Region^c^	RMSPE, %	RSR	MB, %	SB, %	CCC
(36)	NDF_C	13.8 (0.63) + 0.185 (0.0133) × NDF	3,116	Intercontinental	17.0	0.88	0.81	0.04	0.37
				EU	15.1	0.95	3.31	1.04	0.26
				US	20.1	0.99	0.14	2.21	0.13
(37)	EE_C	21.8 (0.62) − 0.452 (0.0763) × EE	2,716	Intercontinental	17.8	0.93	1.38	0	0.27
				EU	15.7	0.99	5.39	0.86	0.18
				US	21.0	1.03	0.29	6.44	‐0.01
(38)	Diet_Com_C	15.4 (0.76) − 0.354 (0.0756) × EE + 0.173 (0.0145) × NDF	2,667	Intercontinental	17.0	0.88	0.88	0.05	0.38
				EU	15.1	0.95	3.25	1.35	0.27
				US	20.0	0.99	0.07	1.74	0.13
(39)	MY_C	23.5 (0.53) − 0.123 (0.0076) × MY	3,384	Intercontinental	17.4	0.91	1.95	0.08	0.34
				EU	15.7	0.99	5.85	1.74	0.21
				US	20.3	1.00	0	3.21	0.11
(40)	ECM_C	22.6 (0.55) − 0.082 (0.0079) × ECM	3,384	Intercontinental	17.8	0.92	1.73	0.03	0.29
				EU	15.9	1.00	6.12	1.71	0.18
				US	20.7	1.02	0.14	4.36	0.03
(41)	ECM + Com_C	21.1 (0.77) − 0.105 (0.0081) × ECM + 1.30 (0.077) × MF − 0.952 (0.1667) × MP	3,384	Intercontinental	16.5	0.86	1.39	0	0.42
				EU	15.1	0.95	4.17	1.90	0.30
				US	19.1	0.94	0.01	0.01	0.21
(42)	Animal_no_DMI_C	15.4 (1.08) − 0.291 (0.0733) × EE + 0.144 (0.0141) × NDF − 0.104 (0.0094) × ECM + 1.34 (0.087) × MF − 1.12 (0.187) × MP + 0.00330 (0.000729) × BW	2,566	Intercontinental	16.1	0.84	1.21	0.40	0.49
				EU	14.7	0.93	2.86	2.99	0.37
				US	18.7	0.92	0.15	0.23	0.30

a GEI, gross energy intake (MJ/day); DMI, dry matter intake (kg/day); NDF, dietary neutral detergent fiber concentration (% of DM); EE, dietary ether extract concentration (% of DM); MY, milk yield (kg/day); ECM, energy corrected milk (kg/day); MF, milk fat concentration (%); MP, milk crude protein concentration (%); BW, body weight (kg).

b *n*, number of observations used to construct equations.

c EU, Europe; US, the United States of America; AU, Australia. Number of observations used for model performance cross‐validation: Intercontinental (EU + US + AU; *n* = 2,566); EU (*n* = 1,423); US (*n* = 1,084).

d RMSPE, Root mean square prediction error, expressed as a percentage of observed CH_4_ yield means; RSR, RMSPE‐observations standard deviation ratio; MB, mean bias as a percentage of MSPE, SB, slope bias as a percentage of MSPE; CCC, Concordance Correlation Coefficient.

**Table 6:** Intercontinental CH_4_ intensity prediction (g/kg ECM) prediction equations for various complexity levels and model evaluations across regions

Model development				Model performance^d^					
Equation	Category	Prediction equation^a^	*n* b	Region^c^	RMSPE, %	RSR	MB, %	SB, %	CCC
(43)	GEI_C	15.5 (0.45) − 0.00629 (0.000962) × GEI	3,352	Intercontinental	28.4	0.98	0.06	0.58	0.07
				EU	22.4	1.00	0.70	0.18	0.04
				US	32.3	0.97	1.67	1.58	0.09
(44)	DMI_C	15.5 (0.46) − 0.116 (0.0179) × DMI	3,384	Intercontinental	28.4	0.98	0.05	0.69	0.07
				EU	22.4	0.99	0.72	0.05	0.05
				US	32.4	0.97	1.81	1.60	0.09
(45)	DMI + NDF_C	11.3 (0.73) − 0.103 (0.0190) × DMI + 0.118 (0.0141) × NDF	3,116	Intercontinental	27.5	0.94	0	0.14	0.18
				EU	21.8	0.97	0.01	0.93	0.18
				US	31.7	0.95	1.07	0.48	0.15
(46)	DMI + EE_C	17.7 (0.61) − 0.142 (0.0207) × DMI − 0.462 (0.0820) × EE	2,716	Intercontinental	28.0	0.96	0.05	0.34	0.12
				EU	21.8	0.97	0.33	0.03	0.11
				US	32.1	0.97	1.20	0.14	0.13
(47)	DMI + Com_C	13.2 (0.86) − 0.127 (0.0207) × DMI − 0.393 (0.0823) × EE + 0.114 (0.0156) × NDF	2,667	Intercontinental	27.2	0.93	0	0.22	0.21
				EU	21.4	0.95	0.01	0.40	0.22
				US	31.4	0.95	0.74	0.30	0.18
(48)	DMI + Com_Comp_C	10.5 (0.71) − 0.364 (0.0825) × EE + 0.120 (0.0156) × NDF	2,677	Intercontinental	27.7	0.95	0.01	0.27	0.15
				EU	21.6	0.96	0.04	0.27	0.18
				US	32.3	0.97	1.67	0.43	0.11
(49)	ECM + Com_C	3.72 (0.602) + 2.87 (0.147) × MP	3,384	Intercontinental	26.9	0.92	0	0.60	0.22
				EU	20.7	0.92	0	0.04	0.28
				US	31.1	0.94	0.79	1.33	0.19
(50)	Animal_C	−0.101 (1.0980) − 0.215 (0.0213) × DMI − 0.118 (0.0301) × CP − 0.323 (0.0760) × EE + 0.120 (0.0142) × NDF − 0.253 (0.0901) × MF + 3.44 (0.183) × MP + 0.00947 (0.000836) × BW	2,566	Intercontinental	24.8	0.85	0	0.11	0.42
				EU	19.9	0.88	0.40	1.54	0.42
				US	27.8	0.84	0.04	1.23	0.42
(51)	Animal_no_DMI_C	−2.85 (1.112) − 0.118 (0.0307) × CP − 0.289 (0.0784) × EE + 0.124 (0.0146) × NDF + 3.32 (0.168) × MP + 0.00605 (0.000762) × BW	2,566	Intercontinental	25.6	0.88	0.02	0.27	0.35
				EU	20.2	0.90	0.76	1.31	0.39
				US	29.2	0.88	0.17	2.86	0.31

a GEI, gross energy intake (MJ/day); DMI, dry matter intake (kg/day); NDF, dietary neutral detergent fiber concentration (% of DM); EE, dietary ether extract concentration (% of DM); CP, dietary crude protein concentration (% of DM); MY, milk yield (kg/day); ECM, energy corrected milk (kg/day); MF, milk fat concentration (%); MP, milk crude protein concentration (%); BW, body weight (kg).

b *n*, number of observations used to construct equations.

c EU, Europe; US, the United States of America; AU, Australia. Number of observations used for model performance cross‐validation: Intercontinental (EU + US + AU; *n* = 2,566); EU (*n* = 1,423); US (*n* = 1,084).

d RMSPE, Root mean square prediction error, expressed as a percentage of observed CH_4_ intensity means; RSR, RMSPE‐observations standard deviation ratio; M, mean bias as a percentage of MSPE, SB, slope bias as a percentage of MSPE; CCC, Concordance Correlation Coefficient.

Methane production was predicted by fitting a mixed effect model using the lmer (Bates, Maechler, Bolker, & Walker, 2015) procedure of R statistical language (R Core Team 2016; version 3.3.0). The model was developed as shown in Equation (iv):

(iv) $$Y=\beta_{0}+\beta_{1}X_{1}+\beta_{2}X_{2}+\cdots +\beta_{n}X_{n}+S_{i}\left(R_{j}\right)+R_{j}+\epsilon ,$$

where *Y* denotes the response variable of CH_4_ production (g/day per cow), CH_4_ yield (g/kg DMI) or CH_4_ intensity (g/kg ECM); β_0_ denotes the fixed effect of intercept; *X*_1_ to *X*_*n*_ denote the fixed effects of predictor variables and β_1_ to β_*n*_ are the corresponding slopes; *S*_*i*_(*R*_*j*_) denotes the random study effects nested in research group; *R*_*j*_ denotes the random research group effects (research groups that contributed data for analysis were used to capture variations such as different regional weather conditions, CH_4_ measurement methods used, research protocols etc.); ε denotes the within‐experiment error. Explanatory variables, which play a key role in predicting CH_4_ production were selected for DMI + Com_C, Diet_Com_C, ECM + Com_C, Animal_C, and Animal_no_DMI_C using a comprehensive selection approach as follows: all of the subset models were fitted to the complete data set (total of 2,566 observations) and the corresponding Bayesian information criterion (BIC) scores were computed. The BIC was calculated as: $n\log\frac{{\mathrm{SSE}}_{p}}{n}+\left(\log n\right)\;p$, where *p* is the number of regression coefficients, *n* is the sample size, and SSE_*p*_ is error sum of squares. A model with a smaller BIC is preferred because it reaches a balance between the goodness of fit and model complexity. In addition, the presence of multicollinearity of fitted models was examined conservatively based on variance inflation factor (VIF). A VIF in excess of 5 was considered an indicator of multicollinearity (Kutner et al., 2005), and identified predictor variables with the largest VIF were removed from the model one at a time. Similar variable selection procedures as described above were adopted for CH_4_ yield (for Diet_Com_C, ECM + Com_C, and Animal_no_DMI_C) and CH_4_ intensity (for DMI + Com_C, Diet_Com_C, Milk_Com_C, Animal_C, and Animal_no_DMI_C).

### 2.3 Cross‐validation and model evaluation

The predictive accuracy of fitted CH_4_ prediction models at different categories was evaluated using the revised k‐fold cross‐validation method (James, Witten, Hastie, & Tibshirani, 2014), based on the refined complete data set (total of 2,566 observations), with folds composed of individual study (*k* = number of studies). Each individual fold was treated as a validation set. The prediction of CH_4_ production of each fold was computed using the model that was fitted from the remaining folds as described by Moraes et al. (2014). The predictions of all folds were used to conduct model evaluation metrics as described below. Evaluation of all models developed at each category was assessed on intercontinental, EU, and US complete data sets.

A combination of model evaluation metrics was used to assess model performance including mean square prediction error (MSPE), root MSPE (RMSPE), mean absolute error (MAE), and concordance correlation coefficient (CCC). The MSPE was calculated according to Bibby and Toutenburg (1977) as shown in Equation (v):

(v) $$\text{MSPE}=\frac{\sum_{i=1}^{n}{\left(Y_{i}-{\hat{Y}}_{i}\right)}^{2}}{n}$$

where *Y*_*i*_ denotes the observed value of the response variable for the *i*th observation, ${\hat{Y}}_{i}$ denotes the predicted value of the response variable for the *i*th observation, *n* denotes the number of observations. The RMSPE was used to assess overall model prediction accuracy because its output was in the same unit as the observations. In this study, RMSPE was reported as a proportion of observed CH_4_ production means in order to compare the predictive capability of models developed from different data sets. The MAE was calculated as shown in Equation (vi) to quantify the prediction error as suggested by Chai and Draxler (2014):

(vi) $$\text{MAE}=\frac{\sum_{i=1}^{n}\left|Y_{i}-{\hat{Y}}_{i}\right|}{n}$$

In both cases, smaller RMSPE or MAE implies better model performance. The RMSPE to standard deviation of observed values ratio (RSR) was calculated as shown in Equation (vii),

(vii) $$\text{RSR}=\frac{\mathrm{RMSPE}}{S_{o}}$$

where *S*_o_ denotes the standard deviation of observations. It was used to compare the performance of a single model based on data from different regions accounting for the regional variability (Moriasi et al., 2007). Similarly, smaller RSR indicates better model predictive ability given the variability of data. MSPE was decomposed into mean bias (MB) and slope bias (SB) deviations to identify systematic biases. The MB and SB were calculated as shown in Equations (viii) and (ix), respectively:

(viii) $$\text{MB}={\left(\overset{¯}{P}-\overset{¯}{O}\right)}^{2}$$

(ix) $$\text{SB}={\left(S_{p}-r\times S_{o}\right)}^{2}$$

where $\overset{¯}{P}$ and $\overset{¯}{O}$ denote the predicted and observed means, *S*_p_ denotes the standard deviation of predicted values, and *r* denotes the Pearson correlation coefficient.

Furthermore, CCC was conducted that includes a bias correction factor (*C*_b_) and *r*, as measurements of accuracy and precision, respectively (Lin, 1989). The CCC was calculated as shown in Equation (ix),

(x) $$\text{CCC}=r\times C_{b}$$

where

$$\begin{array}{cc} C_{b} & ={\left[\left(v+1/v+u^{2}\right)/2\right]}^{-1}\\ v & =S_{o}/S_{p}\\ u & =\left(\overset{¯}{P}-\overset{¯}{O}\right)/{\left(S_{o}S_{p}\right)}^{1/2} \end{array}$$

where $\overset{¯}{P}$, $\overset{¯}{O}$, *S*_o_, and *S*_p_ were defined above, and *v* provides a measure of scale shift, and μ provides a measure of location shift. The CCC evaluates the degree of deviation between the best‐fit line and the identity line (*y* = *x*), therefore, the CCC of a model that is closer to 1, is an indication of better model performance. Similar evaluation approaches were conducted to test the performance of CH_4_ yield and intensity models. Currently, most national enteric CH_4_ inventories are based on models recommended by IPCC (1997, 2006). Therefore, IPCC models were also evaluated on both intercontinental and regional data sets.

## 3 RESULTS

### 3.1 Database

A general description of the data set collated and summary statistics of dietary composition, intake, milk production and composition, BW and CH_4_ emissions are presented in Table 1. In general, EU and US cows had similar DMI. However, DMI of US cows ranged from 3.9 to 35.4 kg/day, which was more variable than EU cows (8.0 to 33.5 kg/day). On average, US cows emitted less CH_4_ compared to EU cows (340 vs. 392 g/day per cow; Table 1). The CH_4_ production observations from the United States had a larger variability [*S*_o_ = 109 g/day per cow and coefficient of variation (CV) = 33%] than from EU [*S*_o_ = 89 g/day per cow and CV = 23%]. Milk production of US cows was greater than that of EU cows, but MF and MP were greater in EU than US cows. Increased MF and MP were the primary factors causing daily ECM of EU cows to be 0.8 kg/day greater than that of US cows. Methane yield (g/kg DMI) and CH_4_ intensity (g/kg ECM) in EU cows were greater than those of US cows by 18% and 6%, respectively. The EU cows had a greater CH_4_ conversion factor (*Y*_m_) compared to US cows (6.4% vs. 5.4%). Regardless of region, the majority of CH_4_ measurements were made using respiration chambers (70%), while 23 and 6% of the observations were measured using the GreenFeed system and SF_6_ tracer technique, respectively.

Most of the US experimental diets (over 91%) included corn silage, alfalfa hay, alfalfa silage, or grass hay as a forage source, but none included pasture. Frequently used concentrate ingredients in the United States were soybean meal, ground corn, and canola meal. In the EU experimental diets, the major forage sources were grass and corn silages, whereas the most frequently used concentrate sources were soybean meal, barley, and wheat. All cows from AU, NZ, and CL in the database were fed pasture‐based diets. Dietary CP concentration was similar for EU and US diets. The mean dietary concentration of EE was slightly greater for EU compared to US diets (3.6% vs. 3.3% of DM, Table 1), and the median of dietary concentration of EE was proportionally greater for EU than US diets (3.5% vs. 3.0% of DM; data not shown). Experimental diets offered to EU cows had greater NDF concentration compared to those offered to US cows (36.6% vs. 33.3% of DM), which is consistent with increased forage:concentrate ratio in EU diets (data not shown). Similarly, the median of dietary concentration of NDF was proportionally greater for EU than US diets (37.7% vs. 32.5% of DM; data not shown).

### 3.2 Models for methane production

#### 3.2.1 Intercontinental models

**Figure 1:**
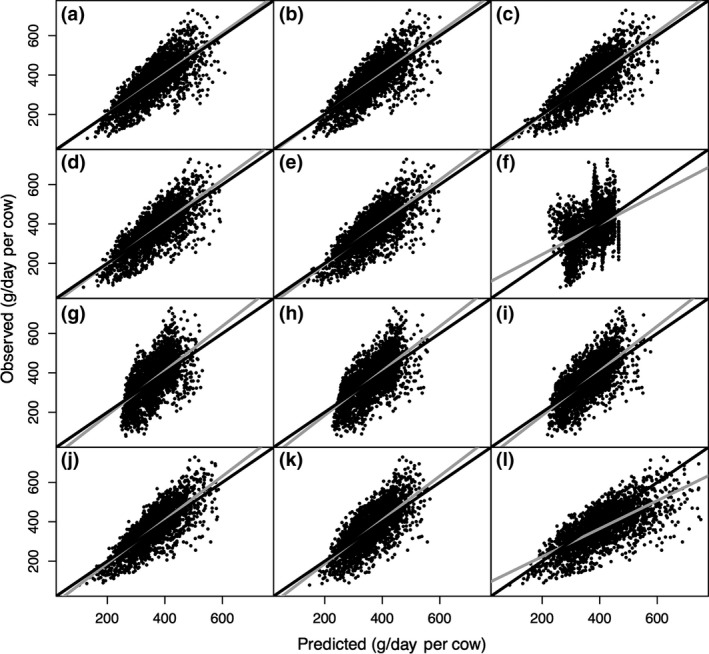
Predicted vs. observed value plots based on Intercontinental CH_4_ production (g/day per cow) prediction equations at different complexity levels of (a) GEI_C (gross energy intake), (b) DMI_C (dry matter intake), (c) DMI + NDF_C (dry matter intake and dietary neutral detergent fiber concentration), (d) DMI + EE_C (dry matter intake and dietary ether extract concentration), (e) DMI + Com_C (DMI and all dietary composition), (f) Diet_Com_C (all available dietary composition only), (g) MY_C (milk yield), (h) ECM_C (energy corrected milk yield), (i) ECM + Com_C (energy corrected milk and milk composition), (j) Animal_C (all available variables), (k) Animal_no_DMI_C (all available variables except DMI), and (l) IPCC Tier 2 (2006) models for lactating dairy cows based on Intercontinental (Europe + US + Australia; *n* = 2,566) data. The corresponding mean absolute errors (MAE, g/day) are MAE_a_ = 50.9, MAE_b_ = 50.3, MAE_c_ = 48.5, MAE_d_ = 51.1, MAE_e_ = 49.2, MAE_f_ = 73.2, MAE_g_ = 62.8, MAE_h_ = 58.9, MAE_i_ = 57.5, MAE_j_ = 47.5, MAE_k_ = 55.1, and MAE_l_ = 64.3. The gray and black solid lines represent the fitted regression line for the relationship between predicted and observed values and the identity line (*y* = *x*), respectively

CH_4_ production prediction equations developed on intercontinental data and model performance indicators are shown in Table 2. As expected, GEI and DMI had a positive linear relationship with CH_4_ production and models based on these variables were of comparable accuracy with negligible bias. Adding dietary NDF to DMI (Equation 3) performed slightly better than one‐variable models or adding dietary EE (Equation 4, Table 2). Although dietary compositions were available for selection in diet based category, only dietary NDF and EE concentrations along with DMI were selected as predictor variables (Equation 5, Table 2), which performed slightly better than those that used only DMI and EE but slightly worse than using DMI and NDF. Dietary NDF was positively correlated with CH_4_ production, while dietary EE had a negative relationship with CH_4_ production. When DMI was excluded, the resulting model (Equation 6) performed worse than any of the previous models. Using ECM and milk composition improved model performance compared with the equation that used MY only. All models using only milk production and composition variables tended to slightly under‐predict at the higher end of production and overpredict at the low end of production (Figure 1). The best overall performance was when DMI, NDF, EE, MF, and BW were selected as predictors (Equation 10; RMSPE = 16.6%). Taking DMI out of the potential variables selected showed reduced prediction performance indicating that DMI is a key variable in predicting CH_4_ production. The error decomposition of overall systematic bias remained negligible regardless of model complexity. Evaluation through CCC and MAE across different model categories was in agreement with RMSPE. Animal_C and DMI + NDF_C models had the largest CCC (0.76 and 0.75, respectively; Table 2) and the smallest MAE (47.5 and 48.5 g/day, respectively; Figure 1).

The predictive ability of intercontinental models on regional data set (EU and US) was also evaluated using RSR. The intercontinental models had a larger RSR (averaging 0.73) on EU observations compared to using all data (averaging 0.70). A greater amount of systematic biases (both MB and SB) was observed with CH_4_ prediction for EU cows than for all cows when using intercontinental models (average 8% vs. 2%, respectively). The predictive ability of intercontinental models on US observations was similar to the overall evaluation, and systematic biases were also similar (Table 2). The most recent IPCC Tier 2 model (IPCC, 2006) performed well on EU data with a low RMSPE (16.2%) and moderate SB (9.6%). The older IPCC Tier 2 model (IPCC, 1997) had a better performance on intercontinental and US data compared to IPCC (2006), but was marginally worse on EU data. Both IPCC models had a less favorable prediction performance for US cows compared to almost all equations developed on the intercontinental data, whereas it was marginally worse for EU cows, in part because the equations were developed on the current data.

#### 3.2.2 Regional models (EU)

**Figure 2:**
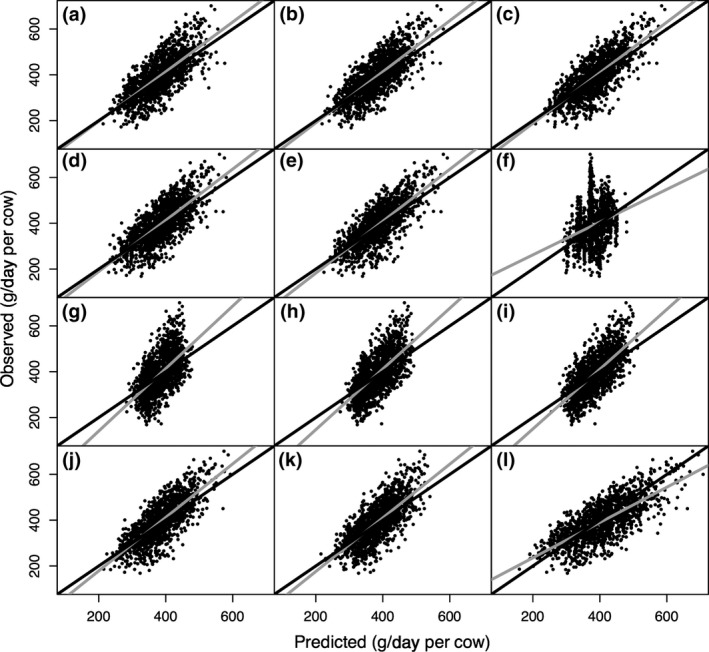
Predicted vs. observed value plots based on European CH_4_ production (g/day per cow) prediction equations at different complexity levels of (a) GEI_C (gross energy intake), (b) DMI_C (dry matter intake), (c) DMI + NDF_C (dry matter intake and dietary neutral detergent fiber concentration), (d) DMI + EE_C (dry matter intake and dietary ether extract concentration), (e) DMI + Com_C (DMI and all dietary composition), (f) Diet_Com_C (all available dietary composition only), (g) MY_C (milk yield), (h) ECM_C (energy corrected milk yield), (i) ECM + Com_C (energy corrected milk and milk composition), (j) Animal_C (all available variables), (k) Animal_no_DMI_C (all available variables except DMI), and (l) IPCC Tier 2 (2006) models for lactating dairy cows based on European (*n* = 1,423) data. The corresponding mean absolute errors (MAE, g/day) are MAE_a_ = 48.6, MAE_b_ = 46.3, MAE_c_ = 44.9, MAE_d_ = 46.3, MAE_e_ = 44.6, MAE_f_ = 65.8, MAE_g_ = 56.1, MAE_h_ = 52.7, MAE_i_ = 51.6, MAE_j_ = 44.5, MAE_k_ = 50.0, and MAE_l_ = 50.7. The gray and black solid lines represent the fitted regression line for the relationship between predicted and observed values and the identity line (*y* = *x*), respectively

Models developed on the EU database and model evaluations are presented in Table 3. The internal EU model evaluations based on EU observations and model comparisons across different categories followed a trend similar to the intercontinental prediction models. Adding NDF to DMI improved model accuracy compared to using either DMI or GEI alone or adding EE to DMI (Table 3). A model with dietary concentrations without DMI did not perform as well as models in previous categories. Models using ECM and milk composition performed better than those using MY only. When all predictors were available for selection, DMI, dietary EE, dietary NDF, MF, and BW were selected and had a similar performance (RMSPE = 14.6%, Equation 23) as the DMI + NDF. Once again, if DMI was taken out, prediction accuracy became worse (RMSPE = 15.8%, Equation 24). Similar to RMSPE, evaluation through CCC and MAE also indicated that models using DMI + NDF and all variables had better prediction accuracy compared to the other models (CCC = 0.72 and 0.72, respectively; Table 3) and (MAE = 44.9 and 44.5 g/day, respectively; Figure 2). In addition, the intercontinental and EU models had similar overall performance for predicting enteric CH_4_ production of EU cows (mean = 0.73 and 0.72, respectively). However, systematic biases were proportionally smaller for EU models compared to intercontinental models (4% vs. 8%, respectively). Furthermore, all categories of models based on the EU database had smaller RSR (mean = 0.72) when used to predict CH_4_ production in EU cows compared to prediction for US cows (mean = 0.80). There was significant MB when EU models were evaluated against US data (Table 3).

#### 3.2.3 Regional models (US)

**Figure 3:**
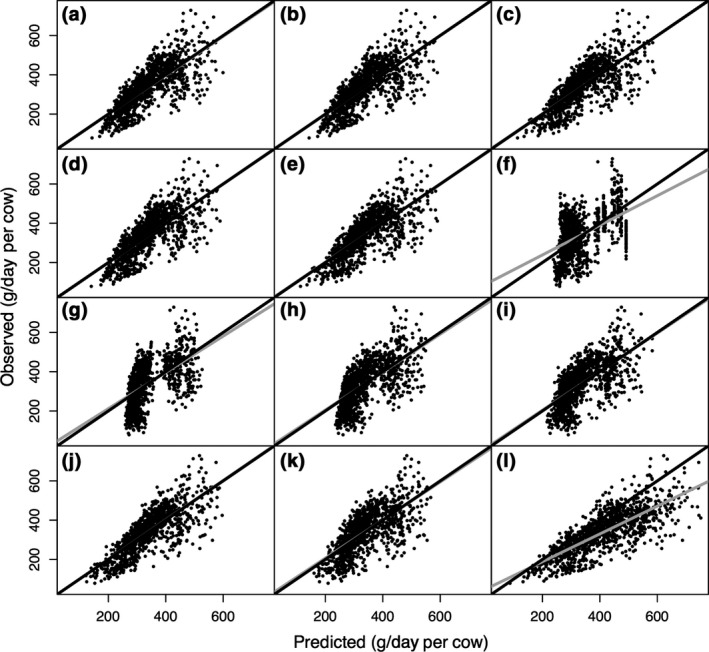
Predicted vs. observed value plots based on US CH_4_ production (g/day per cow) prediction equations at different complexity levels of (a) GEI_C (gross energy intake), (b) DMI_C (dry matter intake), (c) DMI + NDF_C (dry matter intake and dietary neutral detergent fiber concentration), (d) DMI + EE_C (dry matter intake and dietary ether extract concentration), (e) DMI + Com_C (DMI and all dietary composition), (f) Diet_Com_C (all available dietary composition only), (g) MY_C (milk yield), (h) ECM_C (energy corrected milk yield), (i) ECM + Com_C (energy corrected milk and milk composition), (j) Animal_C (all available variables), (k) Animal_no_DMI_C (all available variables except DMI), and (l) IPCC Tier 2 (2006) models for lactating dairy cows based on US (*n* = 1,084) data. The corresponding mean absolute errors (MAE, g/day) are MAE_a_ = 55.1, MAE_b_ = 56.4, MAE_c_ = 55.1, MAE_d_ = 56.9, MAEe = 55.1, MAE_f_ = 78.3, MAE_g_ = 72.5, MAE_h_ = 67.6, MAE_i_ = 65.8, MAE_j_ = 51.7, MAE_k_ = 62.4, and MAE_l_ = 83.6. The gray and black solid lines represent the fitted regression line for the relationship between predicted and observed values and the identity line (*y* = *x*), respectively

Models developed on US data and model evaluations are shown in Table 4. Single variable models using GEI (Equation 25) or DMI (Equation 26) had similar predictive ability when evaluated using US observations, and systematic biases were negligible in both models. Only DMI and dietary NDF concentration were selected from all diet composition, which provided the same prediction equation as the DMI + NDF model (Equations 27 and 29). In contrast to intercontinental and EU based models, CH_4_ production prediction accuracy of US cows was not improved by the addition of any dietary composition in the model. ECM and milk component based models had smaller RMSPE (24.8% and 24.3%, respectively) compared to the MY only based model (26.5%). Consistently, the model containing the most variables also had the smallest RMSPE across all categories (19.8%, Equation 34; Table 4), and had the greatest CCC (0.77) and the smallest MAE (51.7 g/day; Figure 3). When DMI was not considered as a candidate in the prediction equation, ECM was selected instead in the model (Equation 35). Models without DMI had greater RMSPE compared to other categories with DMI in the model. The intercontinental models had similar overall performance for predicting enteric CH_4_ production of US observations compared to US models (mean RSR = 0.71 vs. 0.71, respectively), as both were associated with negligible systematic biases (Table 4). All categories of US models performed better when predicting CH_4_ production from US cows compared with predicting for EU cows (mean RSR = 0.71 vs. 0.82, respectively). Significant increment on both MB and SB were observed when predicting CH_4_ production of EU cows using models based on the US data.

### 3.3 Models for methane yield

Intercontinental CH_4_ yield (g/kg DMI) prediction models of various complexity levels and with evaluations based on different datasets are shown in Table 5. Results for the regional based models of CH_4_ yield are given in Tables S1 and S2 for EU and US, respectively. In both intercontinental and regional models, we observed positive associations between dietary NDF concentration, MF, and BW with CH_4_ yield and negative associations between EE, MY, MP, and ECM with CH_4_ yield. Using only EE, MY or ECM had similar predictive ability for CH_4_ yield (average of these three categories RMSPE = 17.7%, 15.7%, and 20.7% for intercontinental, EU, and US regions, respectively). When all variables were considered, the resultant model had negligible systematic biases and the smallest RMSPE across all categories (RMSPE = 16.1%, Equation 42; Table 5). The CCC and MAE analyses also confirmed that it was the best performing model (Tables 5, S1, and S2). Such findings were also observed in EU and US regional models (Equations 58 and 65, RMSPE = 14.8% and 18.6%, respectively; Tables S1 and S2, respectively). Using milk components as model variables resulted in the second‐best model in all regions. Furthermore, the intercontinental models had a similar RSR while predicting EU or US observations (mean = 0.97 and 0.98, respectively), compared to predicting CH_4_ yield using EU and US regional models (Figures [Link], [Link], [Link]).

### 3.4 Models for methane intensity

Intercontinental CH_4_ intensity (g/kg ECM) prediction models of various complexity levels and with model evaluations based on different datasets are shown in Table 6, and results for the regional models for EU, and the US are shown in Tables S3 and S4, respectively. We consistently observed negative relationships between GEI, DMI, and dietary EE concentration with CH_4_ intensity, and positive relationships between MP, BW and dietary NDF concentration with CH_4_ intensity. However, models that were based on GEI, DMI, or dietary composition did not predict CH_4_ intensity well. Substantial improvement in prediction accuracy was observed when milk component and animal variables were included in the model. Similar to CH_4_ production and yield models, intercontinental models performed well for both EU and US cows (Figures [Link], [Link], [Link]). Models that included the most variables had the greatest CCC and the smallest MAE compared to all other categories in all regions.

## 4 DISCUSSION

### 4.1 Key predictor variables for methane emission

This study identified key predictor variables for CH_4_ production (g/day per cow), yield (g/kg DMI), and intensity (g/kg ECM) in lactating dairy cows from different regions of the world and evaluated the trade‐off between the availability of input variables and prediction accuracy of models. The analysis confirmed that DMI is the most important variable to predict enteric CH_4_ production in dairy cattle, which agrees with previous research (e.g., Hristov et al., 2013; Kriss, 1930; Reynolds, Crompton, & Mills, 2011). There was a significant positive relationship between DMI and CH_4_ production demonstrating that as a dairy cow consumes more feed, more CH_4_ is produced due to greater availability of substrate for microbial fermentation. The majority of extant prediction models for CH_4_ production included DMI as a predictor variable, and evaluation of models developed in this study across various complexity levels also indicated that DMI had the greatest effect on the amount of CH_4_ produced. The slopes of DMI to CH_4_ production ranged from 13.0 to 15.3 g of CH_4_/kg of DMI for EU cows (Table 3) when other covariates were kept constant. The corresponding values were smaller for US cows and ranged from 11.3 to 12.3 g of CH_4_/kg of DMI (Table 4). This is probably due to the difference in dietary composition between EU and US diets and the digestibility of these diets, as EU diets contained proportionally more forage. Practically, it is unlikely that one variable (e.g., dietary NDF concentration) would be different while the rest remain constant because of the associated exchange for other nutrients in ingredients used to formulate diets. In addition, the slopes can only be interpreted in combination with the intercept in all equations. Nevertheless, these results provide insights in assessing the impact of explanatory factors on the variability of CH_4_ production among different regions. Increased intake may potentially increase passage rate and shorten digesta retention time in the rumen, thus decreasing rumen fermentation and organic matter digestibility, which ultimately decrease CH_4_ production per unit of feed (Boadi, Benchaar, Chiquette, & Masse, 2004). Methane yield has been reported to have a negative relationship with DMI (Moe & Tyrrell, 1979). Johnson and Johnson (1995) reported that for every kg of increase in DMI, there is, on average, a 1.6% decrease of feed GE lost through CH_4_. A more recent study also confirmed 2.1% reduction on *Y*_m_ per kg of DMI increase from dairy cows (Warner, Bannink, Hatew, van Laar, & Dijkstra, 2017). Therefore, it is important to use different *Y*_m_ values depending on level of production, which accounts for intake and digestibility of nutrients. In the present study, DMI was not considered as a predictor for CH_4_ yield, and MY or ECM was not used for prediction of CH_4_ intensity because these variables already have been used for the calculation of reported CH_4_ yield or intensity. However, negative relationships between CH_4_ yield and MY (or ECM), and between CH_4_ intensity and DMI were observed because of the overall positive relationship between MY and DMI, which is in close agreement with previous reports (e.g., Johnson & Johnson, 1995; Moe & Tyrrell, 1979; Warner et al., 2017).

In agreement with Kebreab et al. (2008) and Appuhamy et al. (2016), IPCC (2006), the Tier 2 model overpredicted CH_4_ production of US cows in our database by 22%, whereas it performed well on EU cows mainly because the *Y*_m_ in IPCC Tier 2 (6.5%) was similar to the average *Y*_m_ of EU cows (6.4%) in our database. There was a moderate SB of the IPCC (2006) Tier 2 model for EU cows probably due to the absence of an intercept in the IPCC model. The *Y*_m_ for US cows in our database (5.4%) was close to that reported by Kebreab et al. (2008) and Appuhamy et al. (2016) for US cows. This illustrates that it is important to either use a regional model or intercontinental model that was developed using representative samples from each region. Furthermore, the GEI‐based models appear to perform better and are associated with small systematic biases when they include an intercept term as for the GEI_C models developed in the present study.

Dietary NDF concentration was selected previously as the key dietary variable to predict enteric CH_4_ production of dairy cows across regions (Moe & Tyrrell, 1979; Nielsen et al., 2013). Dietary NDF, the majority of which is from forage, represents the amount of structural carbohydrates in the diet. The type of carbohydrates (structural or non‐structural) in the diet has been shown to influence volatile fatty acid (VFA) profile in the rumen, and in turn, enteric CH_4_ production (Russell & Wallace, 1997; Van Soest, 1994). Studies focused on the effect of type of carbohydrates indicate that diets rich in non‐structural carbohydrates such as starch and sugars are more likely to favor propionate formation, resulting in less hydrogen (H) and CH_4_ production, whereas diets rich in structural carbohydrates generally favor acetate and butyrate production by net H producers (Bannink et al., 2008; Johnson & Johnson, 1995; Moe & Tyrrell, 1979). Consistent with effects of digestion and fermentation kinetics on CH_4_ emission, coefficients of NDF in all models developed were significantly positive in the present study. Methane production ranged from 2.80 to 3.42 g of CH_4_/% of dietary NDF for EU cows, when the other covariates were kept constant. The corresponding values were smaller for US cows and ranged from 2.30 to 2.59 g of CH_4_/% of dietary NDF (excluding categories without DMI term). Such findings could result from dietary differences between EU and US. The forage quality in the diet, specifically fiber digestibility, plays an important role in enteric CH_4_ production (Brask, Lund, Hellwing, Poulsen, & Weisbjerg, 2013; Warner et al., 2016), and it has been shown that CH_4_ production of cows tends to increase with increasing diet organic matter digestibility (Ramin & Huhtanen, 2013). The EU diets containing more forage have an inherently greater digestibility of NDF than more concentrate‐based US diets where the lower ruminal pH in the high grain diets inhibits the growth of methanogens and protozoa, in turn, hampering NDF digestion and CH_4_ production in the rumen (Hegarty, 1999). In agreement with Ramin and Huhtanen (2013), Appuhamy et al. (2016) also reported that prediction models for CH_4_ production with digestible dietary NDF concentration had a better performance than the model with dietary NDF concentration in US cows. This indicated that there is potential to further enhance prediction accuracy if dietary acid detergent fiber concentration or apparent total‐tract digestibility of NDF is known. Besides the effect of dietary NDF concentration on total CH_4_ production, cows fed high NDF concentration diets tend to produce more CH_4_ per unit of DMI which can also result from the higher ruminal pH (Knapp, Laur, Vadas, Weiss, & Tricarico, 2014; Pinares‐Patiño, Waghorn, Hegarty, & Hoskin, 2009). Furthermore, substituting high‐fiber forage for the optimal amount of more digestible carbohydrate or low fiber sources will increase milk production, and decrease ruminal pH and fiber digestibility, and both lead to a reduction in CH_4_ intensity (Boadi et al., 2004; Leng, 1993). Consistent with expectations, both enteric CH_4_ yield and intensity declined as dietary NDF concentration decreased in the present evaluation.

Dietary EE concentration was also identified as a key dietary predictor variable in EU and intercontinental enteric CH_4_ production prediction models, but its impact on the predictive ability of US models was minimal. Dietary EE concentration may be increased by using herbage in young, leafy stage rather than in more mature, stemmy stage (Warner et al., 2016), or by lipid supplementation of the diet, and is an indicator of the amount of lipid consumed relative to other dietary components (Martin et al., 2016). The effect of lipid supplementation on enteric CH_4_ production has been extensively studied and lipid supplementation is a well‐recognized mitigation strategy as reviewed by several groups (e.g., Beauchemin, Kreuzer, O'Mara, & McAllister, 2008; Knapp et al., 2014; Martin, Morgavi, & Doreau, 2010). Lipids reduce CH_4_ production by suppressing the protozoa and methanogen population in the rumen, decrease NDF digestibility, and reduce the total amount of organic matter fermented, resulting in lower CH_4_ production (Guyader et al., 2014; Machmüller & Kreuzer, 1999; Van Nevel & Demeyer, 1996). Finally, lipids may cause a reduction in DMI, due to their high energy density and effects on gut fill and appetite, which could lead to less CH_4_ production (Allen, 2000; Hollmann et al., 2012). Consistent with the above‐mentioned studies and data summarized by Grainger and Beauchemin (2011), slopes of the EE variable in the EU prediction models were significantly negative. The absence of dietary EE in US models, and small slope when EE was forced in US models, might be due to the relatively lower EE concentration in the US diets. In addition, the magnitude of reduction has not always been consistent, with some studies reporting a 3.5% (Moate et al., 2011) and a 5.6% (Beauchemin et al., 2008) decrease in CH_4_ yield for 1% increase in dietary EE concentration and others reporting an almost negligible CH_4_ reduction (Eugène, Masse, Chiquette, & Benchaar, 2008). As noted above, dietary lipids can have a negative effect on fiber digestibility in the rumen, DMI, and potentially, MY in dairy cows (Hristov et al., 2013). However, such effects may strongly vary among studies, depending on the compensation of increased energy density to the reduction in fiber digestibility and DMI.

Body weight was one of the variables selected for prediction of CH_4_ production. As noted by Smith and Baldwin (1974) and Demment and Van Soest (1985), ruminal volume and weight are proportional to BW of dairy cows. Consequently, smaller animals, with a lower maintenance energy requirement, ingest less feed and have less CH_4_ production (Hristov et al., 2013). In addition, simulations with a dynamic mechanistic model indicated that the DMI/BW ratio is an important factor for CH_4_ production; consuming same amount of feed intake, smaller cows tend to produce less CH_4_ as ruminal passage rate is likely to be faster due to greater DMI/BW ratio (Huhtanen, Ramin, & Cabezas‐Garcia, 2016; Huhtanen, Ramin, & Udén, 2015), which has been shown to reduce CH_4_ yield (Goopy et al., 2014). Therefore, BW could affect DMI and passage rate of ruminal digesta, which will lead to differences in feed digestibility and VFA production, ultimately affecting CH_4_ production. A positive relationship was observed between BW and CH_4_ production in our evaluation, which agrees with previous research (Hristov et al., 2013; Moraes et al., 2014).

### 4.2 Selection of the best models

In the current study, the trade‐off between model complexity and predictive ability has been evaluated. In general, improvement in model goodness‐of‐fit has been reported as the model structure becomes more complex (e.g., Moraes et al., 2014; Santiago‐Juarez et al., 2016). An evaluation of whole‐farm CH_4_ production models demonstrated poor performance and greater systematic biases when equations did not include dietary variables (Ellis et al., 2010). In the present study, models were categorized and different levels of potential predictor variables were sequentially added during the model development process. We observed that accuracy of prediction of CH_4_ production improved in models that include DMI, dietary composition, milk production and composition, and BW. In particular, complex models that used all available variable information consistently improved prediction performance compared to simpler models. Models using only MY or dietary composition were the least accurate. When DMI was omitted from the model to predict CH_4_ production, ECM was selected instead due to its high correlation with DMI, but model predictive ability was reduced. Although intercontinental models were developed based on a data set containing a slightly greater proportion of EU data compared to US data (55% vs. 42%, respectively), the intercontinental models seem to perform well on both regions without significant biases. In addition, the variable inputs required to improve predictions are not always available from commercial dairy farms, for example, DMI and BW of individual dairy cows. Milk yield and milk components are generally available in practice, but CH_4_ production was not predicted well by these input variables. Considering the number of variables required and prediction performance we recommend the equation with DMI + NDF concentration to be used for enteric CH_4_ production. A recent evaluation of extant models using estimated DMI vs. actual DMI measurements indicated enteric CH_4_ emissions could be predicted satisfactorily without DMI measurements for North America, but not for Australia and New Zealand, with accuracy of prediction using Europe data in between (Appuhamy et al., 2016). However, estimation of DMI is still a challenge for dairy farmers in practice because voluntary DMI prediction equations require individual animal information and, in particular BW (Fox, Sniffen, O'Connor, Russell, & Van Soest, 1992; NRC, 2001; Vazquez & Smith, 2000). In this respect, using average or total intake from a group of cows or the whole herd instead of individual measurements could be an alternative for whole‐farm enteric CH_4_ emission estimates, when cows are grouped by milk production, BW, or parity in commercial dairy farms.

Model evaluations across various complexity levels indicated that CH_4_ yield of lactating dairy cows could be predicted successfully with milk production and composition or dietary composition based models. The corresponding intercontinental models were able to make comparable predictions for EU and US cows relative to the regional EU and US models. The best prediction of CH_4_ intensity could be achieved with the most complex model (Animal_C), with the model for the intercontinental data set also able to make accurate predictions for both EU and US cows. Although overall predictive performance was similar, it should be noted that actual predictions based on the model derived from intercontinental data may differ from the model based on regional‐specific data, because the slopes of the variables included in these models differ.

Finally, it is important to note that the majority of data used in this study was from temperate regions and there is a scarcity of dairy cow data from tropical regions, which differ in breeds and the quality of forage fed. Many developing countries are in the tropical regions, and milk production rather than GHG emissions is still the top priority in those countries (FAO, 2016). Therefore, further research on determinants and predictors of CH_4_ emission applicable to animals in the tropics is warranted. In addition, spatial auto correlations should also be considered to incorporate the effects of environmental factors once a broader database becomes available in the future.

In summary, our analysis based on a relatively large dataset from the GLOBAL NETWORK project, indicated that the ability to predict enteric CH_4_ production increases with increasing model complexity. As observed previously, DMI is the key factor for enteric CH_4_ production prediction. Although complex models that use DMI, NDF, EE, MF, and BW had the best performance for predicting CH_4_ production, models requiring only DMI or DMI + NDF had the second best predictive ability and offer an alternative to complex models. Milk production and composition variables are key factors to predict CH_4_ yield, whereas milk composition and animal variables are key factors to predict CH_4_ intensity. Model evaluation specific to individual regions compared with that of intercontinental based models suggests that enteric CH_4_ production, yield, and intensity can be accurately predicted from both intercontinental models and regional‐specific models with similar performance. Although prediction performance was similar, intercepts and slopes of variables in optimal prediction equations developed on intercontinental basis differed from those developed on regional basis. Therefore, revised CH_4_ emission conversion factors for specific regions are preferred to improve CH_4_ production estimates in national inventories.
