# Supplementary figures and images for: Prediction of enteric methane production, yield, and intensity in dairy cattle using an intercontinental database
Source: Glob Chang Biol. 2018 Mar 8;24(8):3368–89. doi: 10.1111/gcb.14094 (PMC6055644; doi:10.1111/gcb.14094)

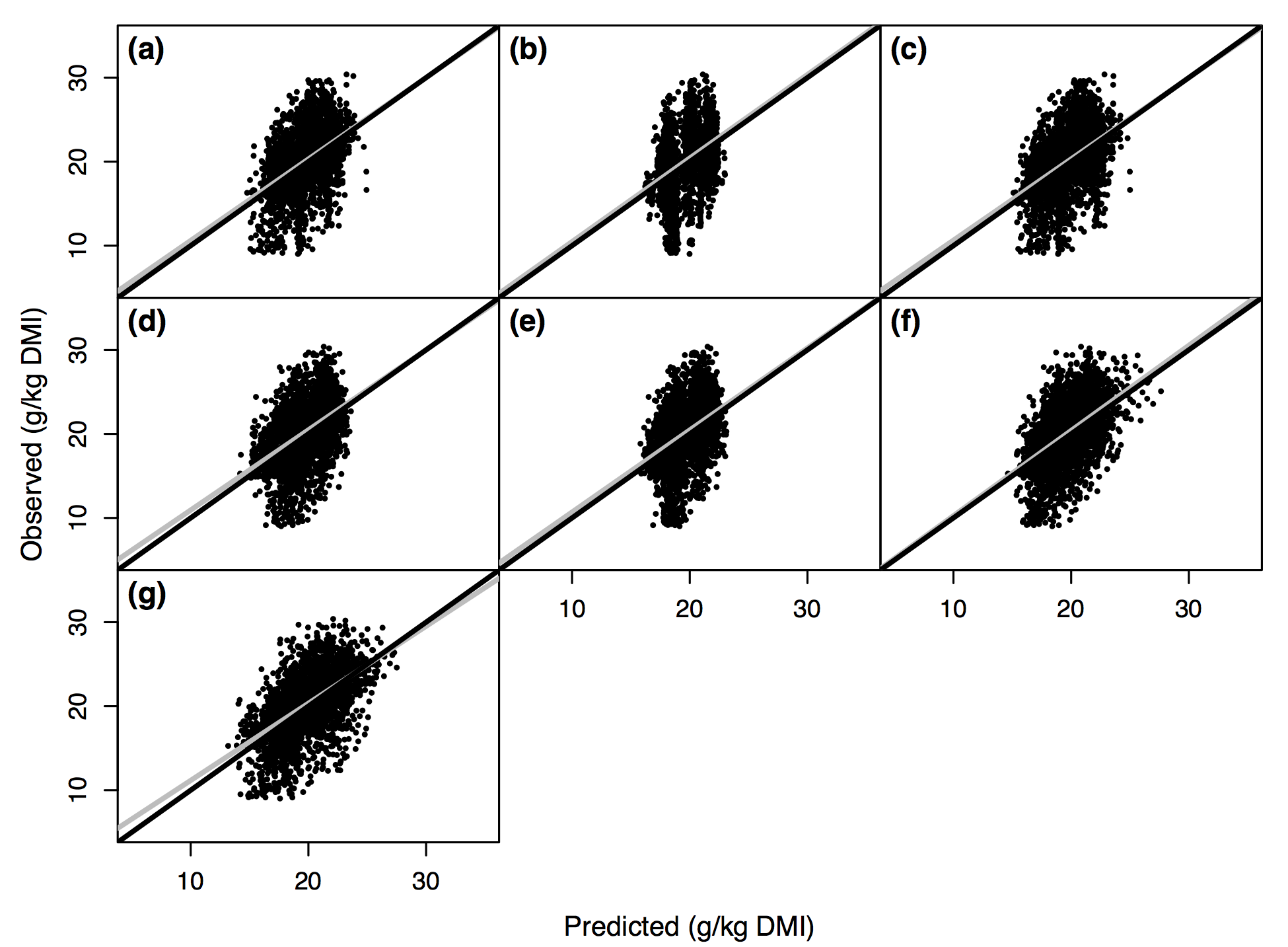

Supplement: Supplementary file 1 [file GCB-24-3368-s001.tiff]

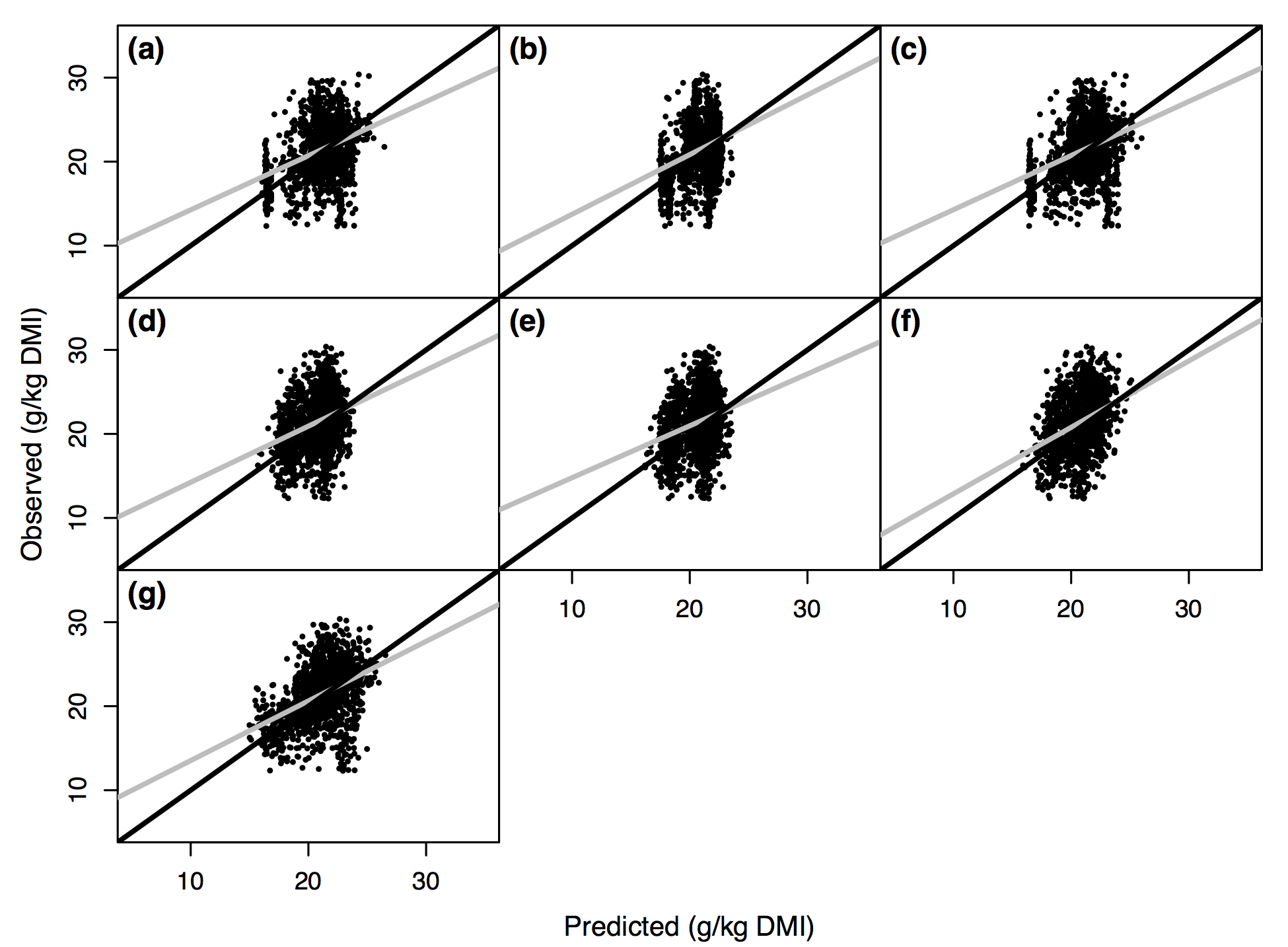

Supplement: Supplementary file 2 [file GCB-24-3368-s002.tiff]

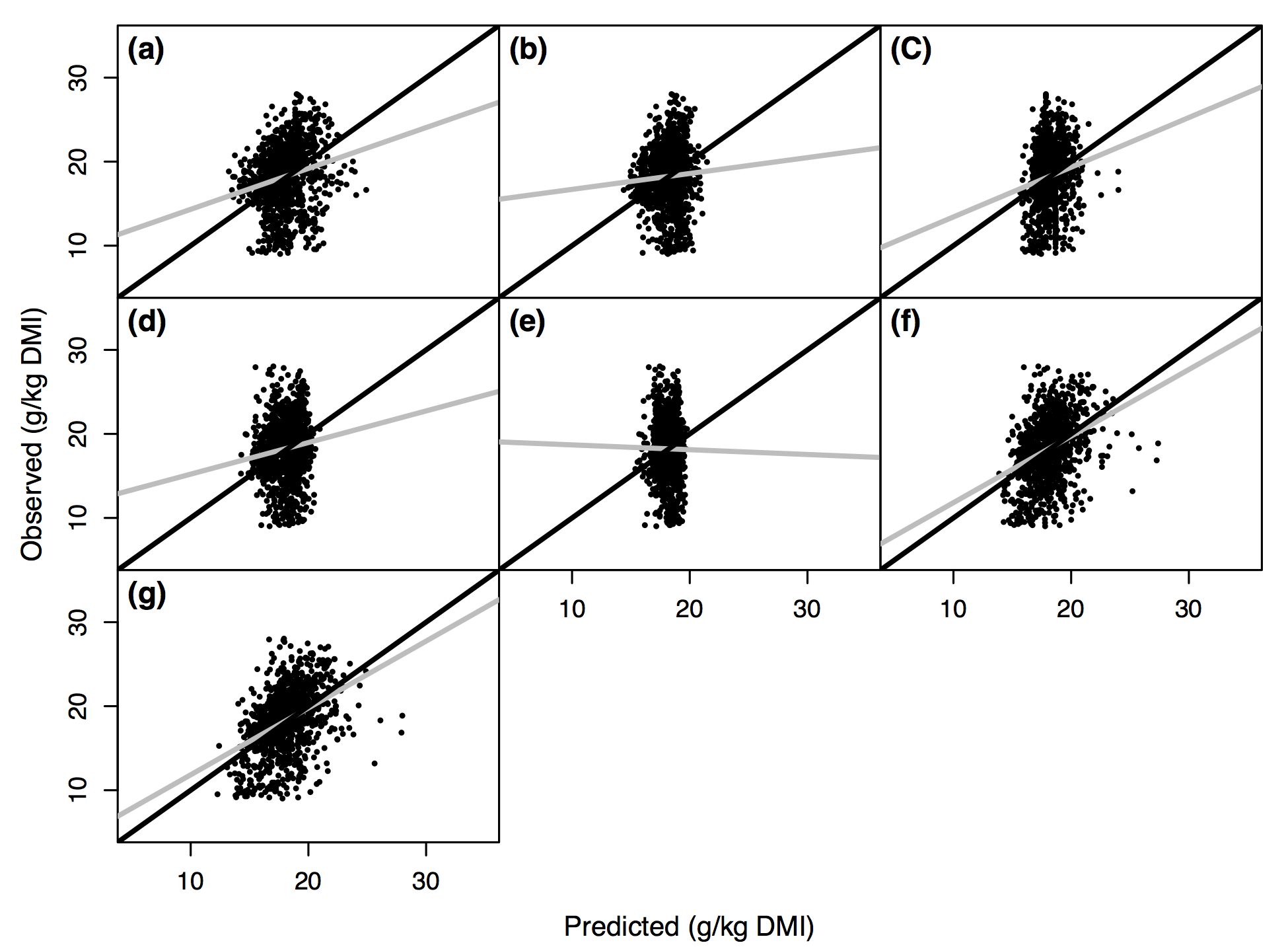

Supplement: Supplementary file 3 [file GCB-24-3368-s003.tiff]

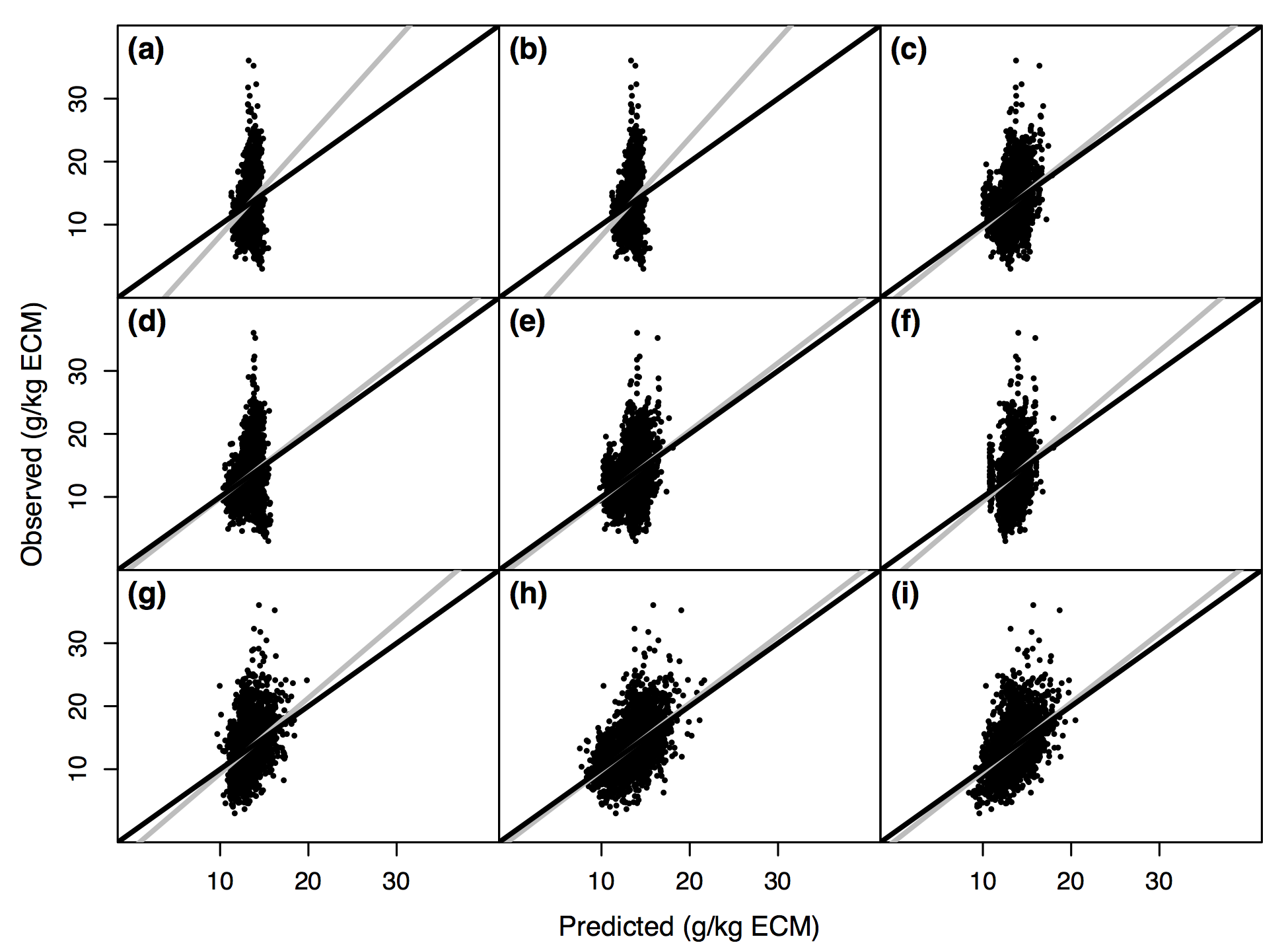

Supplement: Supplementary file 4 [file GCB-24-3368-s004.tiff]

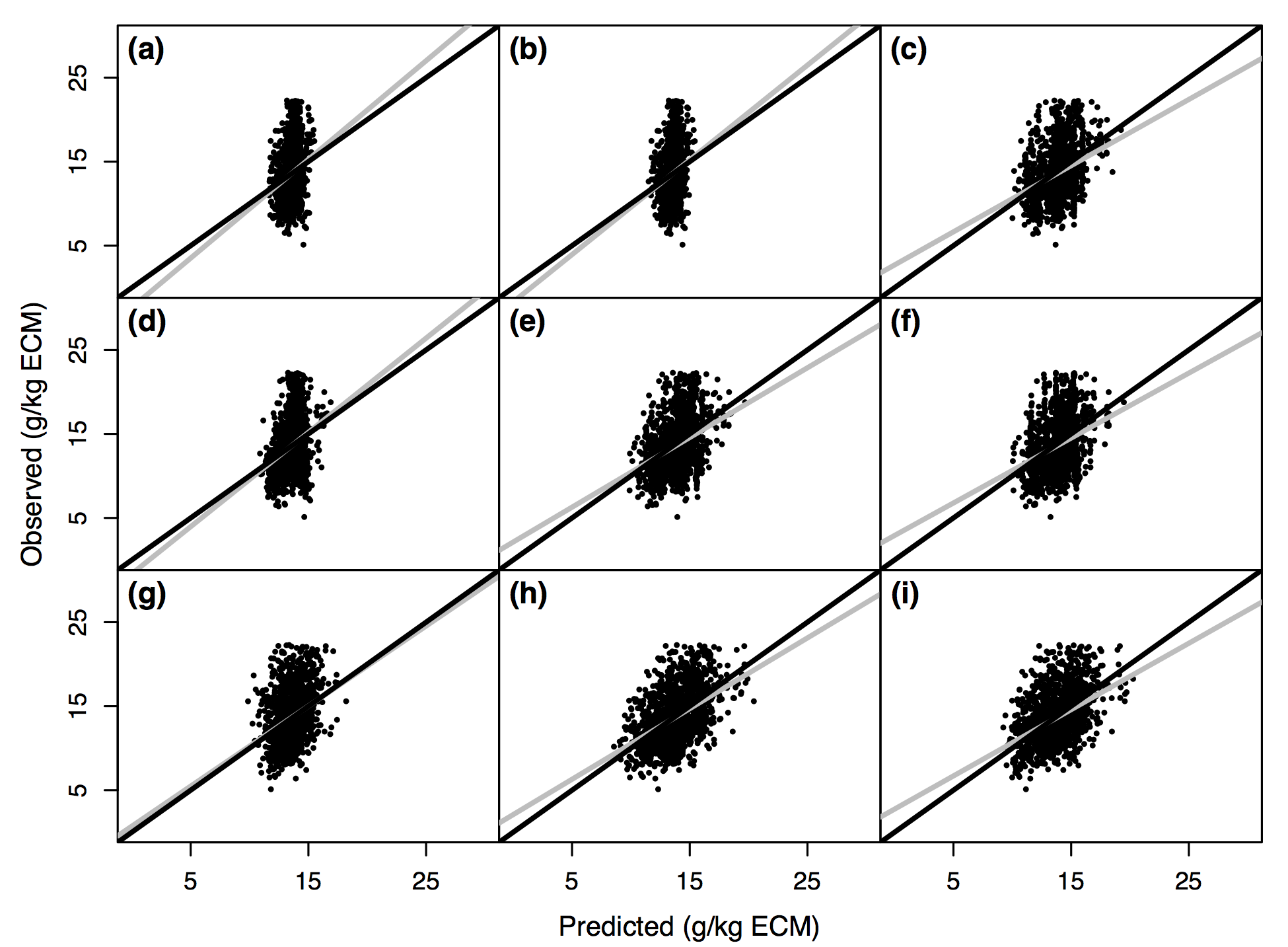

Supplement: Supplementary file 5 [file GCB-24-3368-s005.tiff]

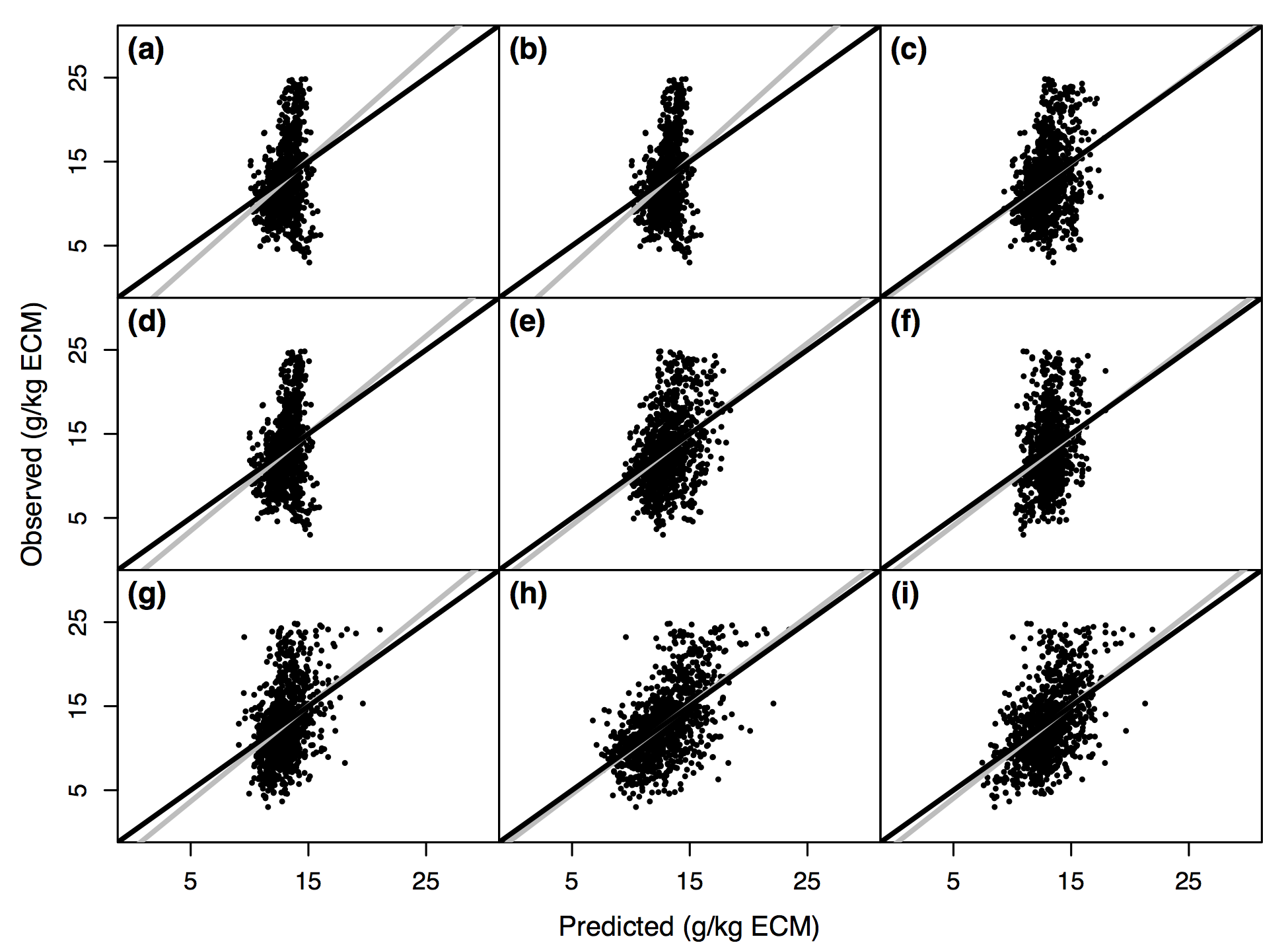

Supplement: Supplementary file 6 [file GCB-24-3368-s006.tiff]
