# Supplementary material for: Prediction of enteric methane production, yield, and intensity in dairy cattle using an intercontinental database
Source: Glob Chang Biol. 2018 Mar 8;24(8):3368–89. doi: 10.1111/gcb.14094 (PMC6055644; doi:10.1111/gcb.14094)
Supplement: Supplementary file 7 [file GCB-24-3368-s007.docx]

Table S1. Europe (EU) CH_4_ yield prediction (g/kg DMI) prediction equations for various complexity levels and model evaluations across regions.

| Model Development | | | |  | Model Performance§ | | | | | |
| --- | --- | --- | --- | --- | --- | --- | --- | --- | --- | --- |
| Eq. | Category | Prediction Equation* | n† |  | Region‡ | RMSPE,  % | RSR | MB,  % | SB,  % | CCC |
| [52] | NDF_C | 12.4 (0.81) + 0.235 (0.0172) × NDF | 1,779 |  | Intercontinental | 18.1 | 1.07 | 0.94 | 2.38 | 0.30 |
|  |  |  |  |  | EU | 15.2 | 4.23 | 0.96 | 0.97 | 0.29 |
|  |  |  |  |  | US | 22.4 | 3.53 | 1.10 | 9.40 | 0.15 |
| [53] | EE_C | 22.7 (0.70) - 0.579 (0.0852) × EE | 1,516 |  | Intercontinental | 19.3 | 1.18 | 1.00 | 3.02 | 0.10 |
|  |  |  |  |  | EU | 15.6 | 1.59 | 0.98 | 3.34 | 0.19 |
|  |  |  |  |  | US | 24.8 | 1.38 | 1.22 | 31.8 | 0.0 |
| [54] | Diet_Com_C | 14.0 (1.01) - 0.375 (0.0857) × EE + 0.224 (0.0205) × NDF | 1,467 |  | Intercontinental | 18.2 | 1.02 | 0.95 | 3.02 | 0.28 |
|  |  |  |  |  | EU | 15.2 | 4.33 | 0.96 | 1.11 | 0.30 |
|  |  |  |  |  | US | 22.8 | 2.58 | 1.12 | 23.3 | 0.15 |
| [55] | MY_C | 23.7 (0.67) - 0.120 (0.0100) × MY | 2,022 |  | Intercontinental | 18.4 | 0.53 | 0.96 | 1.27 | 0.21 |
|  |  |  |  |  | EU | 15.6 | 2.61 | 0.99 | 4.26 | 0.23 |
|  |  |  |  |  | US | 22.9 | 1.71 | 1.13 | 23.0 | 0.11 |
| [56] | ECM_C | 22.9 (0.68) - 0.081 (0.0102) × ECM | 2,022 |  | Intercontinental | 19.0 | 0.93 | 0.99 | 1.89 | 0.13 |
|  |  |  |  |  | EU | 15.9 | 2.93 | 1.00 | 4.27 | 0.19 |
|  |  |  |  |  | US | 23.8 | 0.97 | 1.17 | 27.7 | 0.05 |
| [57] | ECM+Com_C | 19.4 (0.75) - 0.083 (0.0100) × ECM + 0.827 (0.0828) × MF | 2,022 |  | Intercontinental | 17.7 | 0.21 | 0.92 | 1.22 | 0.26 |
|  |  |  |  |  | EU | 15.1 | 1.17 | 0.96 | 3.65 | 0.27 |
|  |  |  |  |  | US | 21.8 | 0.09 | 1.07 | 21.4 | 0.13 |
| [58] | Animal_no_DMI_C | 13.8 (1.22) + 0.127 (0.0383) × CP - 0.379 (0.0867) × EE + 0.211 (0.0200) × NDF - 0.219 (0.0641) × ash - 0.0662 (0.01180) × ECM + 0.561 (0.0928) × MF | 1,423 |  | Intercontinental | 18.0 | 1.23 | 0.94 | 3.90 | 0.32 |
|  |  |  |  |  | EU | 14.8 | 3.48 | 0.94 | 1.39 | 0.35 |
|  |  |  |  |  | US | 23.0 | 2.61 | 1.13 | 27.2 | 0.20 |

*GEI = gross energy intake (MJ/day); DMI = dry matter intake (kg/day); NDF = dietary neutral detergent fiber concentration (% of DM); EE = dietary ether extract concentration (% of DM); ash = dietary ash concentration (% of DM); MY = milk yield (kg/day); ECM = energy corrected milk (kg/day); MF = milk fat concentration (%); MP = milk crude protein concentration (%); BW = body weight (kg).

†n = number of observations used to construct model equations.

‡EU = Europe; US = the United States of America; AU = Australia. Number of observations used for model performance cross-validation: Intercontinental (EU + US + AU; n = 2,566); EU (n = 1,423); US (n = 1,084).

§RMSPE = Root mean square prediction error, expressed as a percentage of CH_4_ yield means; RSR = RMSPE-observations standard deviation ratio; MB = mean bias as a percentage of MSPE, SB = slope bias as a percentage of MSPE; CCC = Concordance Correlation Coefficient.

Table S2. US CH_4_ yield prediction (g/kg DMI) prediction equations for various complexity levels and model evaluations across regions.

| Model Development | | | |  | Model Performance§ | | | | | |
| --- | --- | --- | --- | --- | --- | --- | --- | --- | --- | --- |
| Eq. | Category | Prediction Equation* | n† |  | Region‡ | RMSPE,  % | RSR | MB,  % | SB,  % | CCC |
| [59] | NDF_C | 13.2 (0.92) + 0.151 (0.0230) × NDF | 1,187 |  | Intercontinental | 20.0 | 1.04 | 18.2 | 0.19 | 0.16 |
|  |  |  |  |  | EU | 19.7 | 1.24 | 44.8 | 0.10 | 0.15 |
|  |  |  |  |  | US | 20.4 | 1.00 | 0.16 | 2.18 | 0.07 |
| [60] | EE_C | 18.6 (0.56) - 0.153 (0.1420) × EE | 1,141 |  | Intercontinental | 21.7 | 1.13 | 19.7 | 7.34 | -0.02 |
|  |  |  |  |  | EU | 21.9 | 1.38 | 48.3 | 0.74 | 0.01 |
|  |  |  |  |  | US | 20.8 | 1.03 | 0 | 2.54 | -0.05 |
| [61] | Diet_Com_C | 13.3 (1.07) + 0.152 (0.0229) × NDF | 1,141 |  | Intercontinental | 20.0 | 1.04 | 18.2 | 0.19 | 0.2 |
|  |  |  |  |  | EU | 19.7 | 1.24 | 44.8 | 0.10 | 0.2 |
|  |  |  |  |  | US | 20.4 | 1.00 | 0 | 2.18 | 0 |
| [62] | MY_C | 22.2 (0.91) - 0.119 (0.0120) × MY | 1,212 |  | Intercontinental | 19.7 | 1.02 | 13.5 | 0 | 0.15 |
|  |  |  |  |  | EU | 19.3 | 1.22 | 33.4 | 0.96 | 0.05 |
|  |  |  |  |  | US | 20.5 | 1.01 | 0.13 | 3.45 | 0.09 |
| [63] | ECM_C | 20.6 (0.75) - 0.074 (0.0126) × ECM | 1,212 |  | Intercontinental | 21.9 | 1.14 | 20.9 | 1.54 | 0.01 |
|  |  |  |  |  | EU | 22.3 | 1.41 | 49.6 | 0.29 | 0.01 |
|  |  |  |  |  | US | 20.7 | 1.02 | 0.06 | 4.08 | 0 |
| [64] | ECM+Com_C | 20.6 (1.44) - 0.120 (0.0126) × ECM + 2.15 (0.139) × MF - 1.84 (0.294) × MP | 1,212 |  | Intercontinental | 17.7 | 0.92 | 4.25 | 1.17 | 0.36 |
|  |  |  |  |  | EU | 17.1 | 1.08 | 10.9 | 8.49 | 0.19 |
|  |  |  |  |  | US | 19.1 | 0.94 | 0.06 | 1.19 | 0.27 |
| [65] | Animal_no_DMI_C | 13.3 (1.88) + 0.118 (0.0212) × NDF - 0.130 (0.0137) × ECM + 2.20 (0.144) × MF - 1.71 (0.294) × MP + 0.00521 (0.001207) × BW | 1,084 |  | Intercontinental | 16.5 | 0.86 | 1.94 | 1.12 | 0.48 |
|  |  |  |  |  | EU | 15.4 | 0.97 | 4.78 | 4.72 | 0.33 |
|  |  |  |  |  | US | 18.6 | 0.91 | 0.18 | 1.43 | 0.35 |

*GEI = gross energy intake (MJ/day); DMI = dry matter intake (kg/day); NDF = dietary neutral detergent fiber concentration (% of DM); EE = dietary ether extract concentration (% of DM); MY = milk yield (kg/day); ECM = energy corrected milk (kg/day); MF = milk fat concentration (%); MP = milk crude protein concentration (%); BW = body weight (kg).

†n = number of observations used to construct model equations

‡EU = Europe; US = the United States of America; AU = Australia. Number of observations used for model performance cross-validation: Intercontinental (EU + US + AU; n = 2,566); EU (n = 1,423); US (n = 1,084).

§RMSPE = Root mean square prediction error, expressed as a percentage of CH_4_ yield means; RSR = RMSPE-observations standard deviation ratio; MB = mean bias as a percentage of MSPE, SB = slope bias as a percentage of MSPE; CCC = Concordance Correlation Coefficient.

Table S3. Europe (EU) CH_4_ intensity prediction (g/kg ECM) prediction equations for various complexity levels and model evaluations across regions.

| Model Development | | | |  | Model Performance§ | | | | | |
| --- | --- | --- | --- | --- | --- | --- | --- | --- | --- | --- |
| Eq. | Category | Prediction Equation* | n† |  | Region‡ | RMSPE,  % | RSR | MB,  % | SB,  % | CCC |
| [66] | GEI_C | 15.1 (0.53) - 0.00465 (0.001147) × GEI | 1,990 |  | Intercontinental | 28.1 | 0.97 | 0.06 | 1.83 | 0.09 |
|  |  |  |  |  | EU | 21.9 | 0.97 | 0 | 0.09 | 0.09 |
|  |  |  |  |  | US | 32.3 | 0.97 | 2.42 | 4.47 | 0.09 |
| [67] | DMI_C | 14.8 (0.52) - 0.076 (0.0211) × DMI | 2,022 |  | Intercontinental | 28.3 | 0.97 | 0.07 | 1.75 | 0.08 |
|  |  |  |  |  | EU | 22.0 | 0.98 | 0.01 | 0.05 | 0.08 |
|  |  |  |  |  | US | 32.5 | 0.98 | 2.69 | 5.18 | 0.08 |
| [68] | DMI+NDF_C | 8.35 (0.952) - 0.0470 (0.02297) × DMI + 0.172 (0.0174) × NDF | 1,779 |  | Intercontinental | 27.5 | 0.94 | 0.07 | 0.20 | 0.22 |
|  |  |  |  |  | EU | 21.4 | 0.95 | 0.68 | 0.88 | 0.24 |
|  |  |  |  |  | US | 32.1 | 0.97 | 0.34 | 0.62 | 0.16 |
| [69] | DMI+EE_C | 17.7 (0.72) - 0.124 (0.0247) × DMI - 0.539 (0.0827) × EE | 1,516 |  | Intercontinental | 27.7 | 0.95 | 0.25 | 0.49 | 0.15 |
|  |  |  |  |  | EU | 21.4 | 0.95 | 0 | 0.10 | 0.17 |
|  |  |  |  |  | US | 32.1 | 0.97 | 4.21 | 0.44 | 0.16 |
| [70] | DMI+Com_C | 10.0 (1.16) - 0.0804 (0.02479) × DMI - 0.363 (0.0840) × EE + 0.177 (0.0202) × NDF | 1,467 |  | Intercontinental | 27.0 | 0.93 | 0.18 | 0 | 0.25 |
|  |  |  |  |  | EU | 21.1 | 0.94 | 0.67 | 0.64 | 0.26 |
|  |  |  |  |  | US | 31.4 | 0.95 | 0.91 | 0.03 | 0.21 |
| [71] | Diet_Com_C | 8.03 (0.997) - 0.323 (0.0835) × EE + 0.187 (0.0200) × NDF | 1,467 |  | Intercontinental | 27.5 | 0.95 | 0.20 | 0.10 | 0.21 |
|  |  |  |  |  | EU | 21.4 | 0.95 | 0.66 | 1.06 | 0.24 |
|  |  |  |  |  | US | 32.2 | 0.97 | 1.00 | 0.32 | 0.15 |
| [72] | Milk_Com_C | 5.40 (0.593) - 0.448 (0.0884) × MF + 2.91 (0.179) × MP | 2,022 |  | Intercontinental | 27.3 | 0.94 | 0 | 0.77 | 0.18 |
|  |  |  |  |  | EU | 21.0 | 0.93 | 0.01 | 0.04 | 0.22 |
|  |  |  |  |  | US | 31.5 | 0.95 | 1.12 | 1.11 | 0.16 |
| [73] | Animal_C | - 0.793 (1.2700) - 0.174 (0.0259) × DMI - 0.417 (0.0790) × EE + 0.181 (0.0187) × NDF - 0.602 (0.0970) × MF + 3.05 (0.199) × MP + 0.00762 (0.000898) × BW | 1,423 |  | Intercontinental | 24.9 | 0.86 | 0.02 | 0.14 | 0.41 |
|  |  |  |  |  | EU | 19.8 | 0.88 | 0.09 | 1.11 | 0.42 |
|  |  |  |  |  | US | 28.2 | 0.85 | 0.09 | 0.90 | 0.40 |
| [74] | Animal_no_DMI_C | - 3.48 (1.229) - 0.336 (0.0795) × EE + 0.200 (0.0188) × NDF - 0.577 (0.0984) × MF + 3.12 (0.201) × MP + 0.00477 (0.000802) × BW | 1,423 |  | Intercontinental | 25.9 | 0.89 | 0 | 0.03 | 0.34 |
|  |  |  |  |  | EU | 20.4 | 0.91 | 0.28 | 1.72 | 0.38 |
|  |  |  |  |  | US | 29.8 | 0.90 | 0.21 | 0.78 | 0.30 |

*GEI = gross energy intake (MJ/day); DMI = dry matter intake (kg/day); NDF = dietary neutral detergent fiber concentration (% of DM); EE = dietary ether extract concentration (% of DM); MY = milk yield (kg/day); ECM = energy corrected milk (kg/day); MF = milk fat concentration (%); MP = milk crude protein concentration (%); BW = body weight (kg).

†n = number of observations used to construct model equations.

‡EU = Europe; US = the United States of America; AU = Australia. Number of observations used for model performance cross-validation: Intercontinental (EU + US + AU; n = 2,566); EU (n = 1,423); US (n = 1,084).

§RMSPE = Root mean square prediction error, expressed as a percentage of CH_4_ intensity means; RSR = RMSPE-observations standard deviation ratio; MB = mean bias as a percentage of MSPE, SB = slope bias as a percentage of MSPE; CCC = Concordance Correlation Coefficient.

Table S4. US CH_4_ intensity prediction (g/kg ECM) prediction equations for various complexity levels and model evaluations across regions.

| Model Development | | | |  | Model Performance§ | | | | | |
| --- | --- | --- | --- | --- | --- | --- | --- | --- | --- | --- |
| Eq. | Category | Prediction Equation* | n† |  | Region‡ | RMSPE,  % | RSR | MB,  % | SB,  % | CCC |
| [75] | GEI_C | 15.7 (0.67) - 0.00855 (0.001553) × GEI | 1,212 |  | Intercontinental | 28.4 | 0.97 | 1.04 | 0.18 | 0.10 |
|  |  |  |  |  | EU | 22.6 | 1.00 | 3.17 | 0.25 | 0.07 |
|  |  |  |  |  | US | 31.9 | 0.96 | 0.30 | 0.34 | 0.12 |
| [76] | DMI_C | 15.6 (0.65) - 0.154 (0.0292) × DMI | 1,212 |  | Intercontinental | 28.4 | 0.97 | 1.08 | 0.27 | 0.09 |
|  |  |  |  |  | EU | 22.6 | 1.00 | 3.42 | 0.12 | 0.07 |
|  |  |  |  |  | US | 32.0 | 0.96 | 0.33 | 0.39 | 0.12 |
| [77] | DMI+NDF_C | 10.9 (1.05) - 0.148 (0.0295) × DMI + 0.136 (0.0246) × NDF | 1,187 |  | Intercontinental | 27.4 | 0.94 | 0.34 | 0.01 | 0.21 |
|  |  |  |  |  | EU | 21.8 | 0.97 | 0.63 | 1.78 | 0.21 |
|  |  |  |  |  | US | 31.4 | 0.94 | 0.15 | 0.01 | 0.19 |
| [78] | DMI+EE_C | 16.4 (0.81) - 0.160 (0.0317) × DMI - 0.152 (0.1577) × EE | 1,141 |  | Intercontinental | 28.4 | 0.98 | 1.11 | 0.18 | 0.10 |
|  |  |  |  |  | EU | 22.5 | 1.00 | 3.76 | 0.03 | 0.08 |
|  |  |  |  |  | US | 32.1 | 0.97 | 0.39 | 0.15 | 0.12 |
| [79] | DMI+Com_C | 16.7 (1.72) - 0.164 (0.0309) × DMI - 0.275 (0.0731) × CP + 0.114 (0.0256) × NDF | 1,141 |  | Intercontinental | 27.4 | 0.94 | 0.39 | 0.03 | 0.22 |
|  |  |  |  |  | EU | 22.3 | 0.99 | 0.85 | 3.05 | 0.17 |
|  |  |  |  |  | US | 30.6 | 0.92 | 0.24 | 0.11 | 0.25 |
| [80] | Diet_Com_C | 13.9 (1.76) - 0.296 (0.0741) × CP + 0.107 (0.0261) × NDF | 1,141 |  | Intercontinental | 28.2 | 0.97 | 0.17 | 0.10 | 0.13 |
|  |  |  |  |  | EU | 22.5 | 1.00 | 0.56 | 2.68 | 0.11 |
|  |  |  |  |  | US | 31.8 | 0.96 | 0.64 | 0.03 | 0.15 |
| [81] | Milk_Com_C | 0.93 (1.248) + 3.38 (0.289) × MP | 1,212 |  | Intercontinental | 26.8 | 0.92 | 0.82 | 0.20 | 0.26 |
|  |  |  |  |  | EU | 20.7 | 0.92 | 1.98 | 0.28 | 0.31 |
|  |  |  |  |  | US | 30.6 | 0.92 | 0.19 | 0.49 | 0.24 |
| [82] | Animal_C | - 1.48 (1.983) - 0.259 (0.0317) × DMI - 0.308 (0.0673) × CP + 0.140 (0.0234) × NDF + 3.86 (0.285) × MP + 0.0116 (0.00144) × BW | 1,084 |  | Intercontinental | 24.8 | 0.85 | 0.33 | 0.50 | 0.46 |
|  |  |  |  |  | EU | 20.9 | 0.93 | 4.07 | 5.41 | 0.43 |
|  |  |  |  |  | US | 27.2 | 0.82 | 0 | 0.15 | 0.48 |
| [83] | Animal_no_DMI_C | - 3.40 (2.176) - 0.340 (0.0694) × CP + 0.121 (0.0243) × NDF + 3.95 (0.292) × MP + 0.00691 (0.001346) × BW | 1,084 |  | Intercontinental | 25.9 | 0.89 | 0.30 | 0.26 | 0.37 |
|  |  |  |  |  | EU | 21.2 | 0.94 | 0.31 | 5.95 | 0.37 |
|  |  |  |  |  | US | 28.6 | 0.86 | 0.15 | 0.40 | 0.39 |

*GEI = gross energy intake (MJ/day); DMI = dry matter intake (kg/day); NDF = dietary neutral detergent fiber concentration (% of DM); EE = dietary ether extract concentration (% of DM); CP = dietary crude protein concentration (% of DM); MY = milk yield (kg/day); ECM = energy corrected milk (kg/day); MF = milk fat concentration (%); MP = milk crude protein concentration (%); BW = body weight (kg).

†n = number of observations used to construct model equations.

‡EU = Europe; US = the United States of America; AU = Australia. Number of observations used for model performance cross-validation: Intercontinental (EU + US + AU; n = 2,566); EU (n = 1,423); US (n = 1,084).

§RMSPE = Root mean square prediction error, expressed as a percentage of CH_4_ intensity means; RSR = RMSPE-observations standard deviation ratio; MB = mean bias as a percentage of MSPE, SB = slope bias as a percentage of MSPE; CCC = Concordance Correlation Coefficient.

Figure S1. Predicted vs. observed value plots for Intercontinental methane yield [g/kg dry matter intake (DMI)] prediction equations at different complexity levels of (a) NDF_C (dietary neutral detergent fiber concentration), (b) EE_C (dietary ether extract concentration), (c) Diet_Com_C (all available dietary composition only), (d) MY_C (milk yield), (e) ECM_C (energy corrected milk), (f) ECM+Com_C (energy corrected milk and milk composition), and (g) Animal_no_DMI_C (all available variables except DMI) for lactating dairy cows based on Intercontinental (EU, US & AU; n = 2,566) data. The corresponding mean absolute errors (MAE, g/kg DMI) are MAE_a_ = 2.69, MAE_b_ = 2.83, MAE_c_ = 2.67, MAE_d_ = 2.79, MAE_e_ = 2.83, MAE_f_ = 2.65, and MAE_g_ = 2.56. The gray and black solid lines represent the fitted regression line for the relationship between predicted and observed values and the identity line (y = x), respectively.

Figure S2. Predicted vs. observed value plots for European methane yield [g/kg dry matter intake (DMI)] prediction equations at different complexity levels of (a) NDF_C (dietary neutral detergent fiber concentration), (b) EE_C (dietary ether extract concentration), (c) Diet_Com_C (all available dietary composition only), (d) MY_C (milk yield), (e) ECM_C (energy corrected milk), (f) ECM+Com_C (energy corrected milk and milk composition), and (g) Animal_no_DMI_C (all available variables except DMI) for lactating dairy cows based on Europe (n = 1,423) data. The corresponding mean absolute errors (MAE, g/kg DMI) are MAE_a_ = 2.51, MAE_b_ = 2.63, MAE_c_ = 2.50, MAE_d_ = 2.67, MAE_e_ = 2.70, MAE_f_ = 2.58, and MAE_g_ = 2.43. The gray and black solid lines represent the fitted regression line for the relationship between predicted and observed values and the identity line (y = x), respectively.

Figure S3. Predicted vs. observed value plots for US methane yield [g/kg dry matter intake (DMI)] prediction equations at different complexity levels of (a) NDF_C (dietary neutral detergent fiber concentration), (b) EE_C (dietary ether extract concentration), (c) Diet_Com_C (all available dietary composition only), (d) MY_C (milk yield), (e) ECM_C (energy corrected milk), (f) ECM+Com_C (energy corrected milk and milk composition), and (g) Animal_no_DMI_C (all available variables except DMI) for lactating dairy cows based on US (n = 1,084) data. The corresponding mean absolute errors (MAE, g/kg DMI) are MAE_a_ = 2.94, MAE_b_ = 2.97, MAE_c_ = 2.94, MAE_d_ = 2.93, MAE_e_ = 2.95, MAE_f_ = 2.74, and MAE_g_ = 2.67. The gray and black solid lines represent the fitted regression line for the relationship between predicted and observed values and the identity line (y = x), respectively.

Figure S4. Predicted vs. observed value plots for Intercontinental methane intensity [g/kg energy correct milk (ECM)] prediction equations at different complexity levels of (a) GEI_C (gross energy intake), (b) DMI_C (dry matter intake), (c) DMI+NDF_C (dry matter intake and dietary neutral detergent fiber concentration), (d) DMI+EE_C (dry matter intake and dietary ether extract concentration), (e) DMI+Com_C (DMI and all dietary composition), (f) Diet_Com_C (all available dietary composition only), (g) ECM+Com_C (energy corrected milk and milk composition), (h) Animal_C (all available variables), and (i) Animal_no_DMI_C (all available variables except DMI and ECM) for lactating dairy cows based on Intercontinental (Europe + US + Australia; n = 2,566) data. The corresponding mean absolute errors (MAE, g/kg ECM) are MAE_a_ = 2.89, MAE_b_ = 2.89, MAE_c_ = 2.82, MAE_d_ = 2.82, MAE_e_ = 2.76, MAE_f_ = 2.85, MAE_g_ = 2.74, MAE_h_ = 2.50, and MAE_i_ = 2.62. The gray and black solid lines represent the fitted regression line for the relationship between predicted and observed values and the identity line (y = x), respectively.

Figure S5. Predicted vs. observed value plots for European CH_4_ intensity [g/kg energy correct milk (ECM)] prediction equations at different complexity levels of (a) GEI_C (gross energy intake), (b) DMI_C (dry matter intake), (c) DMI+NDF_C (dry matter intake and dietary neutral detergent fiber concentration), (d) DMI+EE_C (dry matter intake and dietary ether extract concentration), (e) DMI+Com_C (DMI and all dietary composition), (f) Diet_Com_C (all available dietary composition only), (g) ECM+Com_C (energy corrected milk and milk composition), (h) Animal_C (all available variables), and (i) Animal_no_DMI_C (all available variables except DMI and ECM) for lactating dairy cows based on Europe (n = 1,423) data. The corresponding mean absolute errors (MAE, g/kg ECM) are MAE_a_ = 2.35, MAE_b_ = 2.37, MAE_c_ = 2.33, MAE_d_ = 2.27, MAE_e_ = 2.28, MAE_f_ = 2.33, MAE_g_ = 2.26, MAE_h_ = 2.12, and MAE_i_ = 2.20. The gray and black solid lines represent the fitted regression line for the relationship between predicted and observed values and the identity line (y = x), respectively.

Figure S6. Predicted vs. observed value plots for US CH_4_ intensity (g/kg ECM) prediction equations at different complexity levels of (a) GEI_C (gross energy intake), (b) DMI_C (dry matter intake), (c) DMI+NDF_C (dry matter intake and dietary neutral detergent fiber concentration), (d) DMI+EE_C (dry matter intake and dietary ether extract concentration), (e) DMI+Com_C (DMI and all dietary composition), (f) Diet_Com_C (all available dietary composition only), (g) ECM+Com_C (energy corrected milk and milk composition), (h) Animal_C (all available variables), and (i) Animal_no_DMI_C (all available variables except DMI and ECM) for lactating dairy cows based on US (n = 1,084) data. The corresponding mean absolute errors (MAE, g/kg ECM) are MAE_a_ = 3.17, MAE_b_ = 3.19, MAE_c_ = 3.12, MAE_d_ = 3.19, MAE_e_ = 3.03, MAE_f_ = 3.22, MAE_g_ = 3.09, MAE_h_ = 2.68, and MAE_i_ = 2.87. The gray and black solid lines represent the fitted regression line for the relationship between predicted and observed values and the identity line (y = x), respectively.
